# Impaired macroglial development and axonal conductivity contributes to the neuropathology of *DYRK1A*-related intellectual disability syndrome

**DOI:** 10.1038/s41598-022-24284-5

**Published:** 2022-11-19

**Authors:** Isabel Pijuan, Elisa Balducci, Cristina Soto-Sánchez, Eduardo Fernández, María José Barallobre, Maria L. Arbonés

**Affiliations:** 1 Instituto de Biología Molecular de Barcelona (IBMB), Spanish National Research Council (CSIC), 08028 Barcelona, Spain; 2 Centro de Investigación Biomédica en Red de Enfermedades Raras (CIBERER), 08028 Barcelona, Spain; 3 Instituto de Bioingeniería, Miguel Hernández University, 03202 Elche, Spain; 4 Centro de Investigación Biomédica en Red de Bioingeniería, Biomateriales y Nanomedicina (CIBER-BBN), 03202 Elche, Spain

**Keywords:** Differentiation, Disease model, Development of the nervous system, Diseases of the nervous system, Glial biology, Neurological disorders

## Abstract

The correct development and activity of neurons and glial cells is necessary to establish proper brain connectivity. *DYRK1A* encodes a protein kinase involved in the neuropathology associated with Down syndrome that influences neurogenesis and the morphological differentiation of neurons. *DYRK1A* loss-of-function mutations in heterozygosity cause a well-recognizable syndrome of intellectual disability and autism spectrum disorder. In this study, we analysed the developmental trajectories of macroglial cells and the properties of the *corpus callosum*, the major white matter tract of the brain, in *Dyrk1a*^+/−^ mice, a mouse model that recapitulates the main neurological features of DYRK1A syndrome. We found that *Dyrk1a*^+/−^ haploinsufficient mutants present an increase in astrogliogenesis in the neocortex and a delay in the production of cortical oligodendrocyte progenitor cells and their progression along the oligodendroglial lineage. There were fewer myelinated axons in the *corpus callosum* of *Dyrk1a*^+/−^ mice, axons that are thinner and with abnormal nodes of Ranvier. Moreover, action potential propagation along myelinated and unmyelinated callosal axons was slower in *Dyrk1a*^+/−^ mutants. All these alterations are likely to affect neuronal circuit development and alter network synchronicity, influencing higher brain functions. These alterations highlight the relevance of glial cell abnormalities in neurodevelopmental disorders.

## Introduction

Autism spectrum disorder (ASD) encompasses a group of neurodevelopmental disorders (NDDs) with a marked genetic component that are defined by impaired social interaction and communication, and stereotypic dysfunctions. These behavioural alterations often coincide with intellectual disability (ID) or developmental delay and language disorders^1^. The heterogeneity in the phenotypic presentation of ASD has for long limited our understanding of the molecular pathways, cell types and neuronal circuits affected in this disorder. However, the identification of genes conferring a risk of ASD in large-scale genomic studies^2–5^ has made it possible to advance in this direction. As such, there is now compelling evidence that alterations to the prenatal and postnatal brain that affect cortical connectivity are pathological drivers of ASD^5–8^.

*DYRK1A* is one of the high-confidence ASD risk genes identified in NDD/ASD genomic studies^3,5,8,9^. Mutations in *DYRK1A* arise de novo, and they include dominant point mutations and small insertions or deletions (indels) that lead to loss-of-function^9–12^. Chromosomal rearrangements and microdeletions affecting one *DYRK1A* allele have also been described in several unrelated patients^13–15^. All these mutations cause characteristic features that define a well-recognizable syndrome of ID/ASD known as MRD7 (Mental Retardation Dominant 7, OMIM: 614104) or DYRK1A-intellectual disability syndrome (DYRK1A syndrome for short, ORPHA: 464306). Core symptoms of DYRK1A syndrome include general developmental delay, microcephaly, moderate to severe ID, speech delay or an absence of communicative language, motor difficulties and a distinctive facial gestalt^10,16,17^. Core symptoms in affected patients are often accompanied by ASD or autistic features and epilepsy^10,17,18^. Although the impact of *DYRK1A* mutations on brain morphology has not been studied systematically, the most frequent magnetic resonance image changes reported in affected cases are cortical atrophy, enlarged ventricles, a small brainstem, delayed myelination and hypoplasia of the *corpus callosum* (CC)^19^.

*DYRK1A* (Dual-specificity Tyrosine phosphorylation Regulated Kinase1A) encodes an evolutionary conserved kinase that regulates several cellular functions and signalling pathways through the phosphorylation of cytosolic and nuclear substrates^20,21^. The functions of this kinase have been studied in the developing and mature central nervous system (CNS) as *DYRK1A* lies on chromosome 21, the triplication of which is critical for the cognitive deficits and other neurological dysfunctions associated with Down syndrome (DS)^22^. In the developing CNS, DYRK1A regulates cell cycle exit of neural progenitors, dendrite and axon development, and physiological apoptosis^22,23^, thereby ensuring proper brain growth and neuronal connectivity. Moreover, *DYRK1A* haploinsufficiency in mice causes the characteristic neurological features of DYRK1A syndrome, including general developmental delay, microcephaly, learning deficits, autism-relevant behaviours, and febrile and tonic–clonic seizures^12,24–26^. The neocortex of haploinsufficient *Dyrk1a*^+/−^ mice is normally laminated^24^ but with a surplus of excitatory neurons and alterations to the proportions of layer-specific neurons, a phenotype that results from early defects in the differentiation of cortical neural stem cells^12,27^. Abnormalities in the basal dendritic arbors of cortical pyramidal neurons have also been reported in *Dyrk1a*^+/−^ mice^28^, suggesting that the defects in connectivity associated with DYRK1A syndrome may arise from alterations in both the timing of cortical neuron production and neuronal morphogenesis.

Astrocytes and oligodendroglial cells are fundamental for the correct assembly, maturation and function of neuronal circuits^29^. There is considerable evidence implicating glial cell dysfunction in ASD pathogenesis^30–32^. Astrocytes and oligodendrocyte precursor cells (OPCs) are generated from gliogenic neural stem cells through stereotypic developmental programs that rely on the interplay between intrinsic and external cues^33–35^. Glial development appears to be affected in DYRK1A syndrome, given the abnormalities in myelination and the CC in children with the syndrome^19^. Indeed, there are alterations to the expression of glial genes and genes involved in gliogenesis in the transcriptome of the early postnatal *Dyrk1a*^+/−^ cerebral cortex^12^. For example, expression of the astrocyte marker glia fibrillary acidic protein (GFAP) is enhanced in regions of the postnatal and young adult *Dyrk1a*^+/−^ mouse brain^24,36^.

Here, the *Dyrk1a*^+/−^ mouse model was used to study the development of cortical glial cells in DYRK1A syndrome. As a result, alterations to astrogliogenesis and OPC production were detected in the developing brain of *Dyrk1a*^+/−^ mice, along with a delay in the progression of the oligodendroglial lineage. Thus, these mice eventually have an excess of cortical astrocytes, hypomyelination of the CC and reduced velocities of action potential (AP) propagation along myelinated and unmyelinated callosal axons. These data indicate that glial cells contribute to the deficits in neuronal connectivity associated with DYRK1A syndrome, and that therapies aimed at promoting oligodendroglial differentiation and myelination may be beneficial for children suffering from this syndrome.

## Results

### Cortical astrogenesis is enhanced in ***Dyrk1a***^+/−^ mice

**Figure 1:**
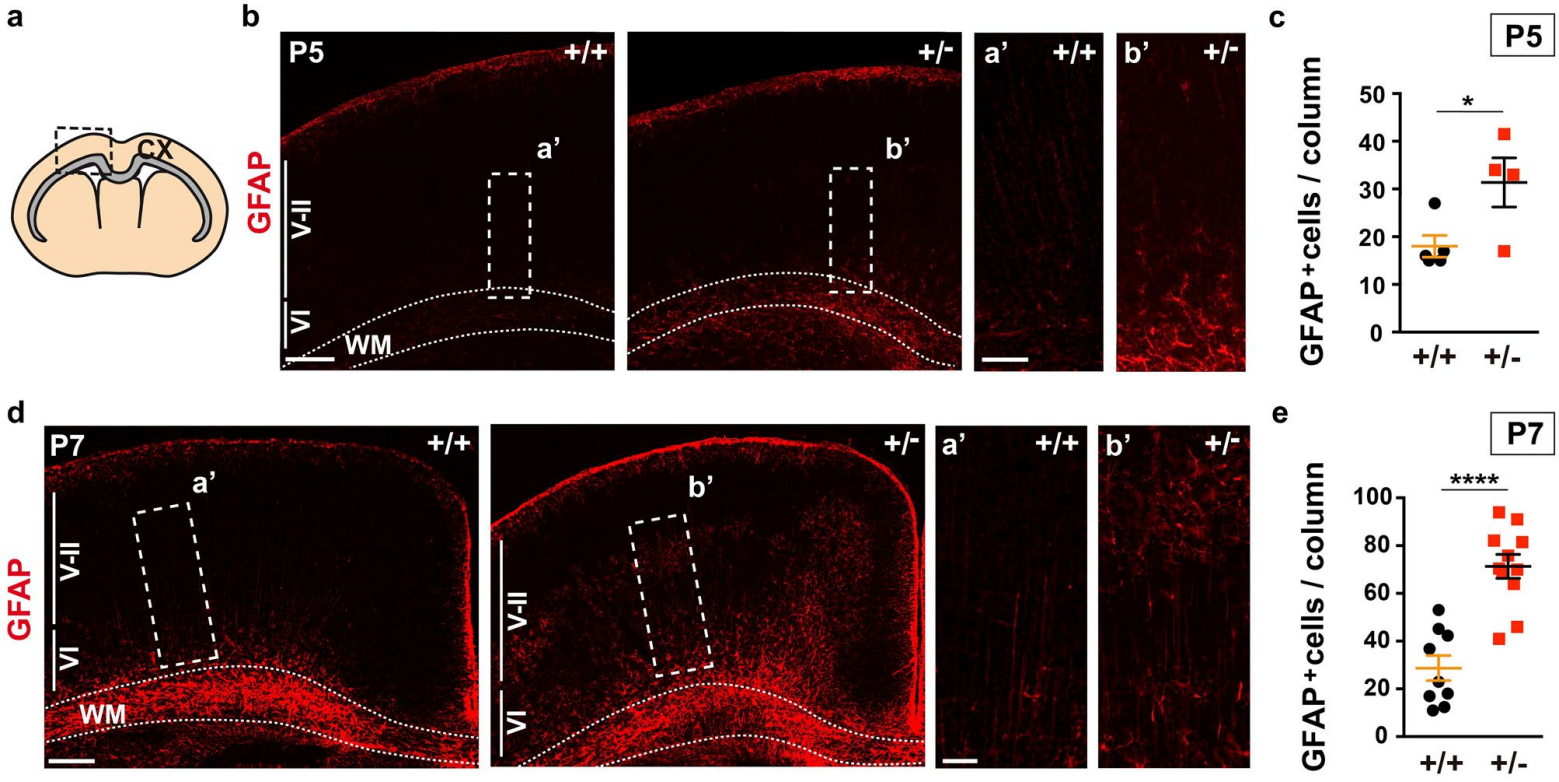
More astrocytes in the postnatal cerebral cortex of *Dyrk1a*^+/−^ mice. (**a**) Schematic representation of a coronal brain section indicating the *corpus callosum* in grey and the region of the cerebral cortex (CX) for cell quantification. (**b**, **d**) Representative brain images from P5 (**b**) and P7 (**d**) *Dyrk1a*^+/+^ (+/+) and *Dyrk1a*^+/−^ (+/−) mice stained for GFAP, indicating the position of the external (V-II) and internal (VI) cortical layers. The boxed areas are magnified to show GFAP^+^ astrocytes. Dotted lines define the white matter (WM). (**c**, **e**) Mean number (± SEM) of GFAP^+^ astrocytes quantified at P5 (**c**) and P7 (**e**) in 1000 μm wide columns expanding layers II to VI. The values correspond to individual animals obtained from 1 litter in **c** and from 2 litters in **e**: *P < 0.05 and ****P < 0.0001, Student’s t-test. Scale bars: 250 μm (**b**); 500 μm (**d**); 100 μm in magnifications.

During development, the neural stem cells of the dorsal telencephalon (also referred to as radial glia cells) first generate the excitatory neurons of the neocortex and subsequently, glial cells. This switch to gliogenesis occurs at around embryonic day (E) 18 in the mouse^33^, with new-born astroglial cells migrating away from the germinal ventricular zone (VZ), thereafter maturing and dividing to populate the entire neocortex^37^. In astrocytes, the expression of GFAP and other protein markers of late differentiation commences during the first week of postnatal life^33^. Thus, cortical astrogenesis was evaluated in haploinsufficient *Dyrk1a*^+/−^ mice by immunostaining the brain for GFAP at postnatal day (P) 5 and P7. GFAP^+^ cells with the characteristic astrocyte staining accumulated in the white matter and cortical layer (I) close to the meninges (Fig. 1a,b,d) of *Dyrk1a*^+/−^ and *Dyrk1a*^+/+^ mice at both developmental stages. A few GFAP^+^ astrocytes were detected in the remaining cortical layers (II to VI) in P5 and P7 control brains, with these cells concentrated in layer (VI), next to the germinal region (Fig. 1b,d). A similar distribution of GFAP^+^ cells to that in the controls was seen in the P5 *Dyrk1a*^+/−^ neocortex but with significantly more cells (Fig. 1c). Astrocytes were more abundant in mutant neocortices at P7 and they were more widespread across the cortical plate than in control animals (Fig. 1d,e). Hence, the production of cortical astrocytes appears to be enhanced in postnatal *Dyrk1a*^+/−^ mutants.

NOTCH and JAK-STAT signalling are key pathways for the initiation of gliogenesis in the developing cerebral cortex^33^. DYRK1A regulates these two pathways in opposite directions, restraining NOTCH signalling by limiting the capacity of its intracellular domain to sustain transcription^38^, while promoting JAK-STAT signalling and enhancing *STAT3* transcriptional activity^39^. The expression of the progliogenic genes *Notch2*, *Sox4*, *Sox11* and *Nfib* was enhanced in the cerebral cortex of new-born *Dyrk1a*^+/−^ mice^12^, suggesting possible defects in the differentiation of the mutant progenitors at the onset of gliogenesis. Thus, the gliogenic potential of dorsal *Dyrk1a*^+/−^ progenitors was evaluated by electroporating the eGFP reporter plasmid into E16.5 *Dyrk1a*^+/+^ and *Dyrk1a*^+/−^ brains, and visualizing the astroglial progeny of the electroporated progenitors in the P5 cerebral cortex immunostained for GFAP (Supplementary Fig. S1a,b). The percentage of cortical GFP^+^ cells that expressed GFAP (GFAP^+^;GFP^+^ cells/total GFP^+^ cells) was similar in the two genotypes (Supplementary Fig. S1c), indicating that *DYRK1A* haploinsufficiency does not compromise the capacity of dorsal progenitors to differentiate into astrocytes. These progenitors were then immunolabeled in E17.5 brains using the progenitor marker SOX2^40^, when cortical neurogenesis is almost complete in both the *Dyrk1a*^+/−^ mutant and control embryos^12^. However, SOX2^+^ cells were more abundant in the dorsal germinal region of *Dyrk1a*^+/−^ than control embryos (Supplementary Fig. S2). Thus, the increase in the number of cortical astrocytes in postnatal *Dyrk1a*^+/−^ mutants (Fig. 1) does not seem to be a result of an increased capacity of the progenitors to differentiate into astrocytes but rather, to an accumulation of progenitors at the end of the neurogenic period.

**Figure 2:**
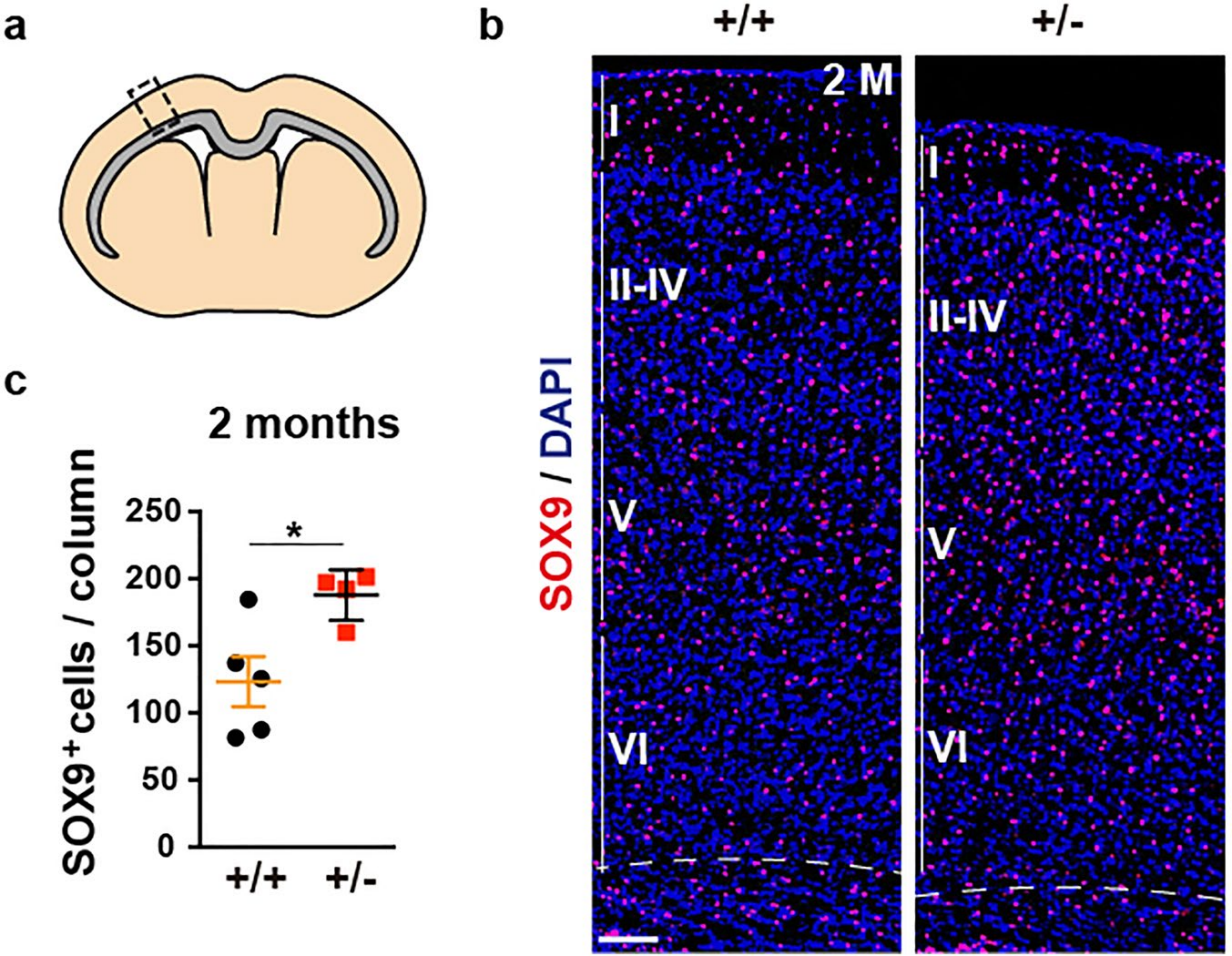
Increased numbers of astrocytes in the neocortex of adult *Dyrk1a*^+/−^ mice. (**a**) Schematic representation of a coronal brain section indicating the *corpus callosum* in grey and the region of the cerebral cortex for cell quantification (rectangle). (**b**) Brain sections from 2-month-old (2 M) *Dyrk1a*^+/+^ (+/+) and *Dyrk1a*^+/−^ (+/−) mice stained for SOX9 and with the nuclei labelled by DAPI (blue). The position of the neocortical layers I to VI are indicated. (**c**) The number of SOX9^+^ astrocytes (mean ± SEM) in 500 μm wide columns. The values correspond to individual animals obtained from 2 litters: *P < 0.05, Student’s t-test. Scale bar: 100 μm.

Accordingly, we then assessed whether the abnormal number of astrocytes in *Dyrk1a*^+/−^ pups was maintained in young adult (P60) animals. GFAP expression in healthy brains varies across regions and this expression is undetectable in many astrocytes by immunohistochemistry, or difficult to attribute to individual cells^41^. Therefore, astrocytes were labelled in adult samples using antibodies against the SOX9 transcription factor expressed by all brain astrocytes outside the neurogenic regions^42^. In *Dyrk1a*^+/+^ and *Dyrk1a*^+/−^ cerebral cortices SOX9^+^ cells were distributed similarly through all layers, although they were more abundant in the mutant neocortex (Fig. 2). A similar increase in SOX9^+^ cells was observed in the *Dyrk1a*^+/−^ hippocampus (Supplementary Fig. S3a-c), consistent with the reportedly higher density of GFAP^+^ cells in the hippocampus of *Dyrk1a*^+/−^ mutants^36^. Microglia also influence neural circuit development^29^ and a dysfunction of these cells has been implicated in ASD^30^. However, when brain microglia were labelled with the cell marker IBA1 (ionized calcium binding adapter molecule 1^43^), no difference in the number of hippocampal or neocortical IBA1^+^ microglial cells was seen between control and *Dyrk1a*^+/−^ mutants, or in their IBA1 staining (Supplementary Figs. S3d,e and S4). Together, these results suggest that microglia do not contribute to the brain phenotype in DYRK1A syndrome and that the abnormal number of astrocytes in the *Dyrk1a*^+/−^ mice was not the result of pathological gliosis but rather, it reflects a developmental problem.

### The cerebral cortex of ***Dyrk1a***^+/−^ mice is deficient in ventral oligodendrocyte progenitor cells

**Figure 3:**
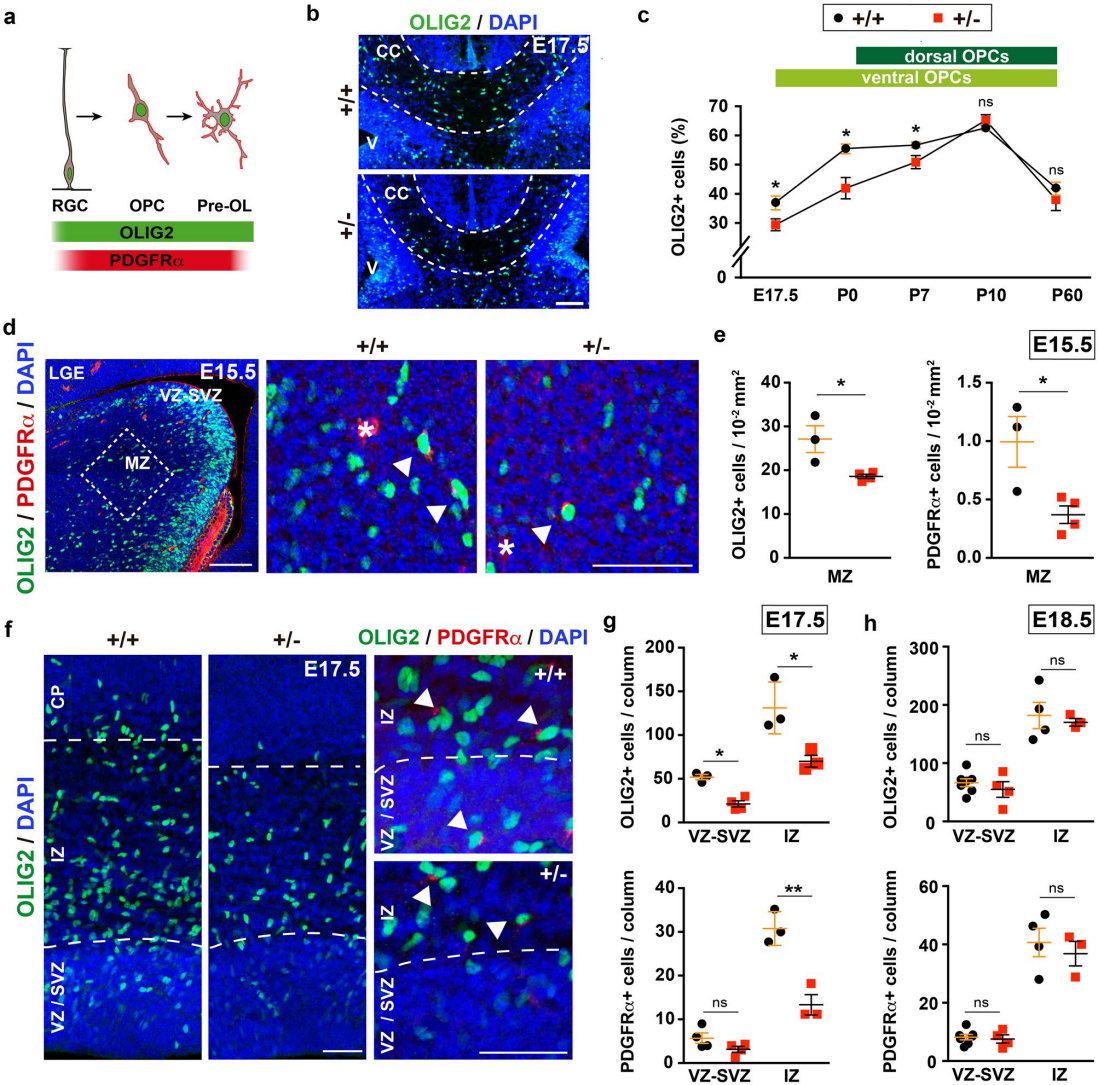
A deficit in ventral oligodendroglia cells during the development of *Dyrk1a*^+/−^ mutants. (**a**) Scheme of progression along the oligodendroglial lineage from a radial glia progenitor (RGC) to a pre-myelinated oligodendrocyte (OL): OPC, oligodendroglial precursor cell. (**b**) Representative images of the central region of the *corpus callosum* (CC: defined by dashed lines) of *Dyrk1a*^+/+^ (+*/*+*)* and *Dyrk1a*^+/−^ (+*/−*) E17.5 brains stained for OLIG2 and with the nuclei labelled by DAPI (blue): V, ventricle. (**c**) Percentage (mean ± SEM) of OLIG2^+^ cells (OLIG2^+^ cells/total cells) in this region at the developmental stages indicated. The ventral and dorsal origins of the OLIG2^+^ cells at these times is indicated. n = 3–9 embryos/animals each condition (1–3 litters each developmental stage). (**d**) Image of the lateral ganglionic eminence (LGE) of a control (+/+) E15.5 brain stained for OLIG2 and PDGFRα, and with the nuclei labelled by DAPI (blue), and magnifications of the region in the mantle zone (MZ, indicated by a dashed box) in a +/+ and +/− brain. Arrowheads indicate OLIG2^+^;PDGFRα^+^ oligodendroglial cells and the asterisks PDGFRα^+^ blood vessel cells: VZ-SVZ, ventricular and subventricular zones. (**e**) Mean density (± SEM) of OLIG2^+^ cells and OLIG2^+^;PDGFRα^+^ (PDGFRα^+^) cells in the MZ region indicated in d (dashed box) of E15.5 +/+ and +/− embryos. (**f**) Images of the dorsal telencephalon of E17.5 +/+ and +/− brains stained for OLIG2 and PDGFRα, and with the nuclei labelled by DAPI (blue) and magnifications of the OLIG2^+^;PDGFRα^+^ cells (arrowheads). (**g**, **h**) The mean number (± SEM) of OLIG2^+^ cells and PDGFRα^+^ cells quantified in the VZ-SVZ or the intermediate zone (IZ) of 200 μm wide columns at E17.5 (**g**) and E18.5 (**h**). Values in e, g and h correspond to individual embryos obtained from 1 to 3 litters: ns, not significant; *P < 0.05; **P < 0.01, Student’s t-test. Scale bar: 100 μm (**b**, **d**) and 50 μm (**f**).

The OPCs that populate the developing cerebral cortex are produced in temporal waves and in different domains of the VZ. In mice, the first OPCs are generated in the ventral telencephalon and they enter the cortex after E16. At birth, another wave of OPC production commences in the dorsal telencephalon, and these dorsal OPCs spread throughout the cortex and reach similar proportions to ventral OPCs by around P10^35,44^. Based on the defects in myelination in children with DYRK1A syndrome and the hypoplasia of the CC (the major white matter tract of the brain)^16,19^, and given the expression of DYRK1A by OPCs in the human (CoDEx database,^45^) and mouse (Supplementary Fig. S5,^46^) brain, oligodendroglial development could also be affected in this syndrome. To assess this possibility, we first counted the cells expressing the oligodendroglial marker OLIG2 in the CC midline of control and *Dyrk1a*^+/−^ mutants at different developmental times (Fig. 3a,b). There was a significantly lower percentage of OLIG2^+^ cells (OLIG2^+^ nuclei/total nuclei) in *Dyrk1a*^+/−^ brain at E17.5 and P0. Moreover, a smaller but significant reduction of OLIG2^+^ cells was also observed at P7 when dorsal OPCs start populating the CC, although OPC numbers reached normal levels three days later (Fig. 3c). The delayed occupation of the mutant CC by OLIG2^+^ cells suggests that ventral OPC production may be impaired. Hence, we examined the onset of oligodendrogenesis (E15.5) in the lateral ganglionic eminence (LGE), the main contributor of ventral originated callosal OPCs^44^. New-born OPCs continue to express OLIG2 as they move from the VZ to the mantle zone and they then begin to express platelet-derived growth factor receptorα (PDGFRα^47^: Fig. 3a). Consistent with a deficit in OPC production, the mantel zone of the *Dyrk1a*^+/−^ LGE contained fewer OLIG2^+^ cells and fewer double labelled OLIG2^+^;PDGFRα^+^ cells than the control LGE (Fig. 3d,e).

Dorsal OPC production was then evaluated in a similar manner but at E17.5, the time at which the first OLIG2^+^ cells appear in the dorsal germinal region. The *Dyrk1a*^+/−^ dorsal telencephalon had fewer OLIG2^+^ cells and fewer OLIG2^+^PDGFRα^+^ OPCs in the germinal region than the controls, as well as fewer OLIG2^+^ cells in the intermediate zone migrating towards the cortical plate (Fig. 3f,g). However, OPC numbers in these cortical regions reached normal levels 24 h later (Fig. 3h), suggesting that OPC production in the dorsal *Dyrk1a*^+/−^ embryonic brain is not impaired but slightly delayed.

### Altered oligodendroglial differentiation in ***Dyrk1a***^+/−^ mutants

**Figure 4:**
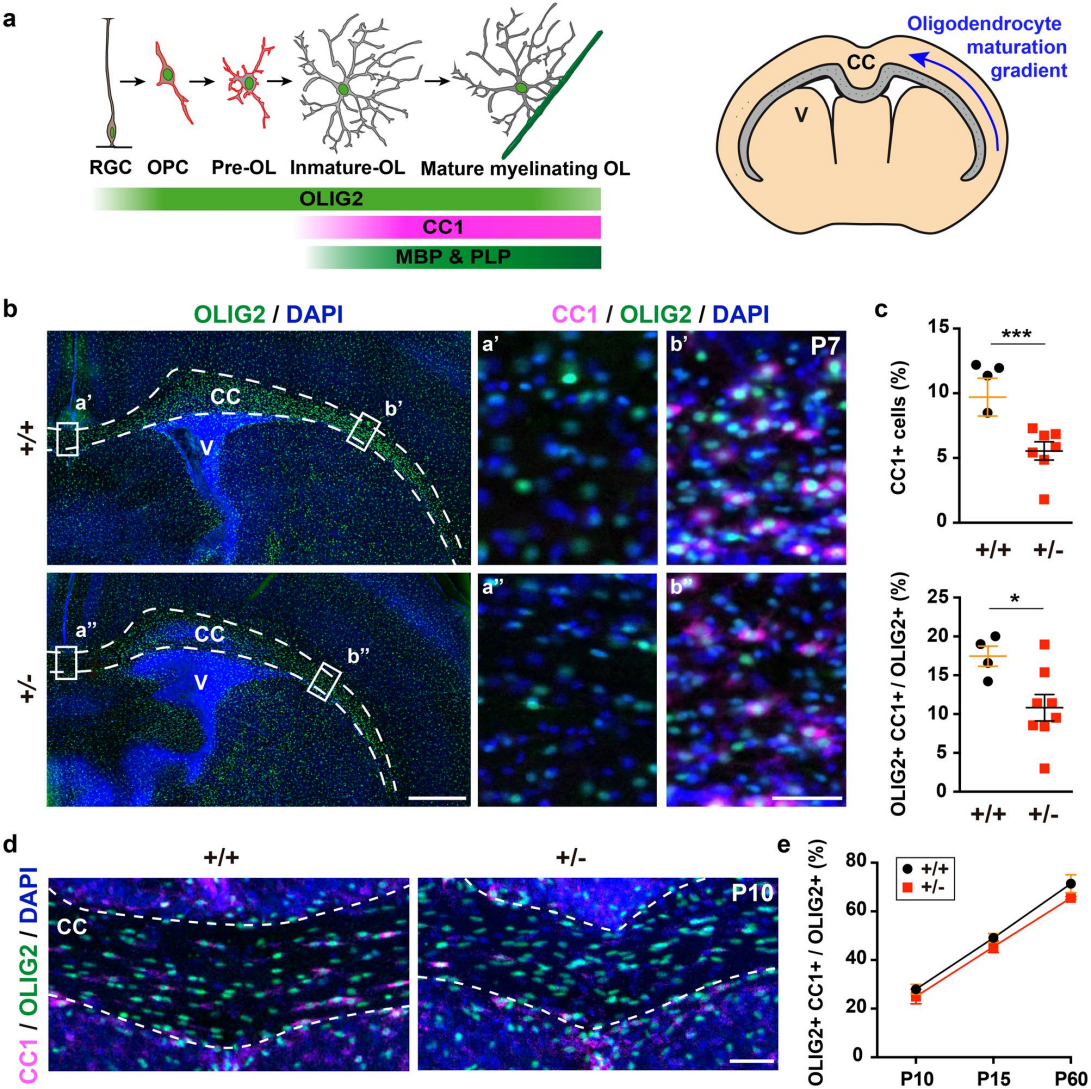
Oligodendroglial differentiation is delayed in the cerebral cortex of postnatal *Dyrk1a*^+/−^ mice. (**a**) Schematic representation of oligodendroglial lineage progression from a radial glial cell (RGC) to a mature myelinating oligodendrocyte (OL), and a coronal brain section indicating the ventral to dorsal gradient of OL maturation in the *corpus callosum* (CC): OPC, oligodendrocyte precursor cell; V, ventricle. (**b**) Representative images of P7 *Dyrk1a*^+/+^ (+*/*+*)* and *Dyrk1a*^+/−^ (+ */*−) coronal brain sections stained for CC1 and OLIG2, and with the nuclei labelled by DAPI (blue). The CC is defined by the dashed lines. Cell counting was performed in the CC regions indicated by rectangles and the images on the right correspond to magnifications of these regions. Note that CC1^+^;OLIG2^+^ cells were only detected in the ventral area (**b’** and **b’’**). (**c**) Percentage (mean ± SEM) of CC1^+^ cells (OLIG2^+^;CC1^+^ cells/total cells) and CC1^+^ oligodendroglial cells (OLIG2^+^;CC1^+^ cells/OLIG2^+^ cells) in these areas. The values represent individual animals obtained from 2 litters: *P < 0.05; ***P < 0.001, Student’s t-test. (**d**, **e**) Representative images of the central region of the CC in P10 + / + and + /- brains stained for CC1 and OLIG2, and with the nuclei labelled by DAPI (blue, **d**). Histogram showing the percentages (mean ± SEM) of CC1^+^ oligodendroglial cells in this area at P10, P15 and P60 (**e**): n = 4–8 animals each condition (1–3 litters each developmental stage). The differences between genotypes were not significant, two way ANOVA. Scale bars: 500 μm (**b**) and 50 μm (**d** and magnifications in **b**).

Progression along the oligodendroglia lineage was examined in the *Dyrk1a*^+/−^ mutant cerebral cortex by analysing the expression of differentiation markers (Fig. 4a). *Dyrk1a*^+/−^ and control P7 brain sections were initially labelled for OLIG2 and the oligodendrocyte marker CC1 (Fig. 4a), the latter expressed in the lateral part of the CC in control brains and diminishing progressively towards the medial region to become almost undetectable at the midline (Fig. 4b). The same lateral to medial expression gradient was observed in the CC of *Dyrk1a*^+/−^ mutants but with a smaller proportion of CC1^+^ cells (CC1 + cells/total cells), and with a smaller population of oligodendroglial cells that expresses CC1^+^ (CC1^+^;OLIG2^+^ cells/total OLIG2^+^ cells) in the mutants than in the controls (Fig. 4c). Moreover, the area of the CC labelled for MBP, a marker of mature myelinating oligodendrocytes (Fig. 4a), was also more restricted in *Dyrk1a*^+/−^ mice at P7 (Supplementary Fig. S6). When the same studies were performed later in development, from P10 to P60, there were no differences between the genotypes in the relative CC1^+^ oligodendrocyte numbers in the centre of the CC (Fig. 4d,e). Hence, oligodendroglial development appears to be delayed in the *Dyrk1a*^+/−^ cerebral cortex.

### Cortical white matter alterations and thinner callosal axons in the ***Dyrk1a***^+/−^ mutant mice

**Figure 5:**
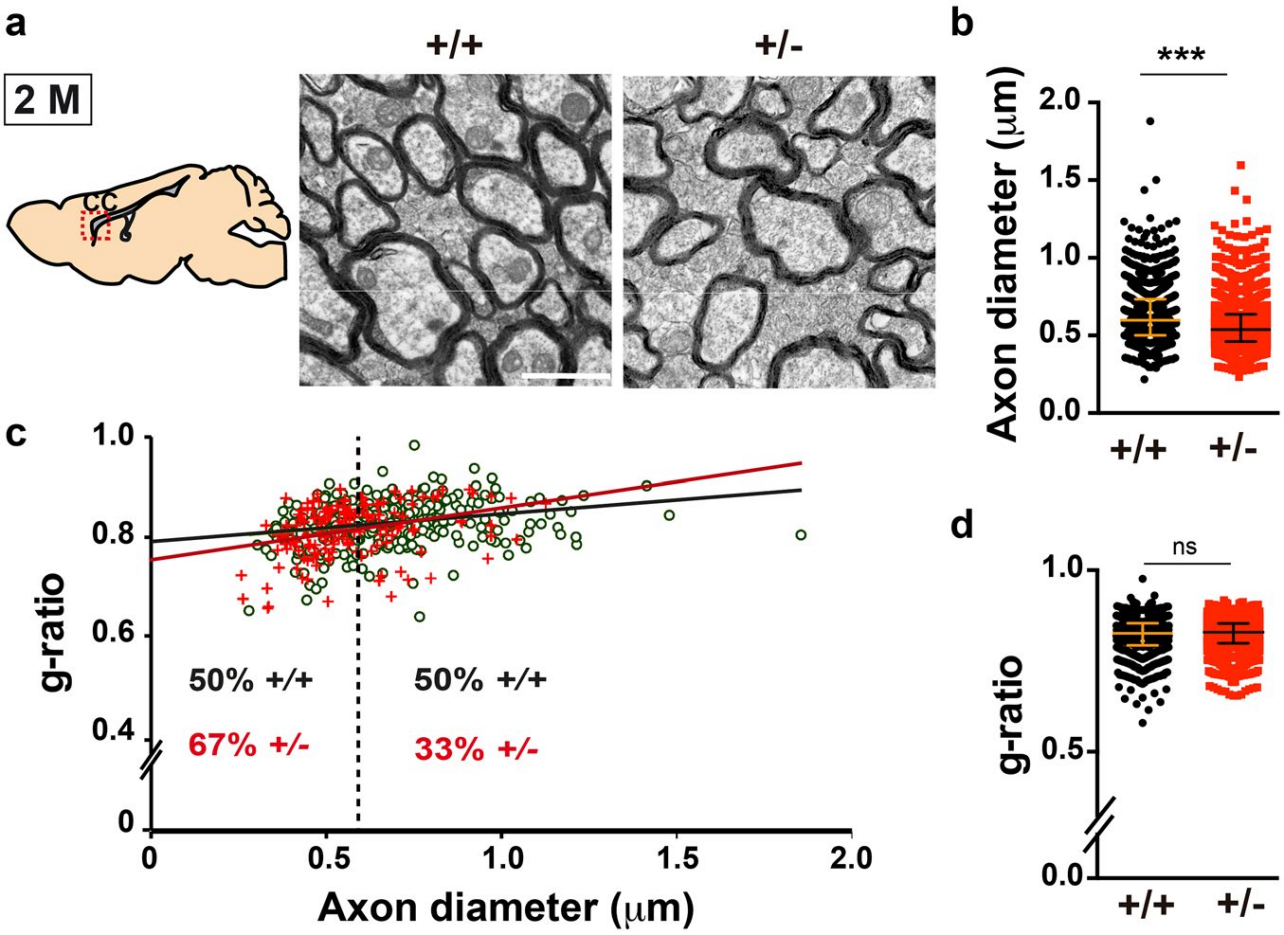
The corpus callosum of adult *Dyrk1a*^+/−^ mice is hypomyelinated. (**a**) Schematic representation of a sagittal brain section indicating the region of the *corpus callosum* (CC) analysed and representative images of the CC ultrastructure in 2-month-old (2 M) *Dyrk1a*^+/+^ (+*/*+) and *Dyrk1a*^+/−^ (+ */*−) mice. Scale bar: 1 μm. (**b**) Median value of the diameter (± interquartile range) of myelinated axons from each genotype. (**c**) Scattered plot of the g-ratios of individual fibres relative to their respective axon diameter. Green dots correspond to +/+ axons and red crosses to +/- axons. The dashed line indicates the median axon diameter in +*/*+ mice and the numbers on both sides of this line the percentages of axons below (left) or above (right) this value for each genotype. (**d**) Median value of the g-ratio (± interquartile range) for each genotype. Values in **b**-**d** were obtained from 980 (+/+) and 2024 (+/−) axons: n = 7–8 animals each genotype (3 litters); ns = not significant; ***P < 0.001, Mann–Whitney U test.

To assess whether the alterations in OPC production and differentiation influence the onset and progression of myelination in *Dyrk1a*^+/−^ mutants, the ultrastructure of the neocortical *Dyrk1a*^+/−^ white matter was analysed at P19, at the onset of myelination (myelination of the CC commences in the second postnatal week and continues at high rates until P45^48^). Electron microscopy showed that there was a similar percentage of myelinated axons in control and *Dyrk1a*^+/−^ mutants (*Dyrk1a*^+/+^ 6.6 ± 0.8%, *Dyrk1a*^+/−^ 4.7 ± 0.9%; *P* = 0.1359, Student’s *t*-test: see Supplementary Fig. S7a), although in the mutants these axons had a smaller calibre and their myelin sheaths were slightly thicker than those around the control axons (smaller g-ratios: axon diameter/fibre diameter: Supplementary Fig. S7b,c). Myelin maturation requires its compaction, and it starts after a few membrane wraps and depends on MBP synthesis^49^. Therefore, the small g-ratios of *Dyrk1a*^+/−^ axons (Supplementary Fig. S7c) could result from a delay in oligodendrocyte differentiation and it correlates with weaker MBP expression. The CC of *Dyrk1a*^+/−^ mice was examined after the rapid phase of myelination, at 2 months of age. At this age, callosal axons in *Dyrk1a*^+/−^ mutants were abnormally packed (No. of total axons/1,000 μm^2^ *Dyrk1a*^+/+^ 2,578 ± 211, *Dyrk1a*^+/−^ 3,433 ± 292; *P* = 0.038, Student’s *t*-test) and the proportion of myelinated axons in the mutant CC decreased significantly (myelinated axons/total axons *Dyrk1a*^+/+^ 53.21 ± 2.27%, *Dyrk1a*^+/−^ 34.48 ± 1.24%; *P* = 0.002, Student’s *t*-test: Fig. 5a). As in P19 animals, myelinated callosal axons were thinner in 2-month-old *Dyrk1a*^+/−^ mice than in the controls (Fig. 5b,c), although the g-ratio values were normal (Fig. 5c,d). This result indicates that myelin-membrane growth is not affected in *Dyrk1a*^+/−^ mutants and that compaction of the growing myelin sheaths would appear to be delayed. As axon diameter is a determinant factor for myelination^50,51^, hypomyelination of the *Dyrk1a*^+/−^ mutant’s CC could result from dysfunctional axon development.

To further characterize the white matter in the *Dyrk1a*^+/−^ cerebral cortex the expression of MBP and PLP, two major myelin-associated proteins in the CNS, was analysed in 2-month-old mice. At this age, similar cortical expression of *Mbp* and *Plp1* (the gene encoding PLP) was evident in *Dyrk1a*^+/+^ and *Dyrk1a*^+/−^ mice, along with similar levels of MBP isoforms. However, the cerebral cortex of *Dyrk1a*^+/−^ mutants accumulated twice as much PLP than the controls (Supplementary Fig. S8, original blots are presented in Supplementary Fig. S10), suggesting alterations in myelin biogenesis and/or PLP trafficking to the oligodendrocyte plasma membrane.

### ***Dyrk1a***^+/−^ mutants have abnormal nodes of Ranvier

**Figure 6:**
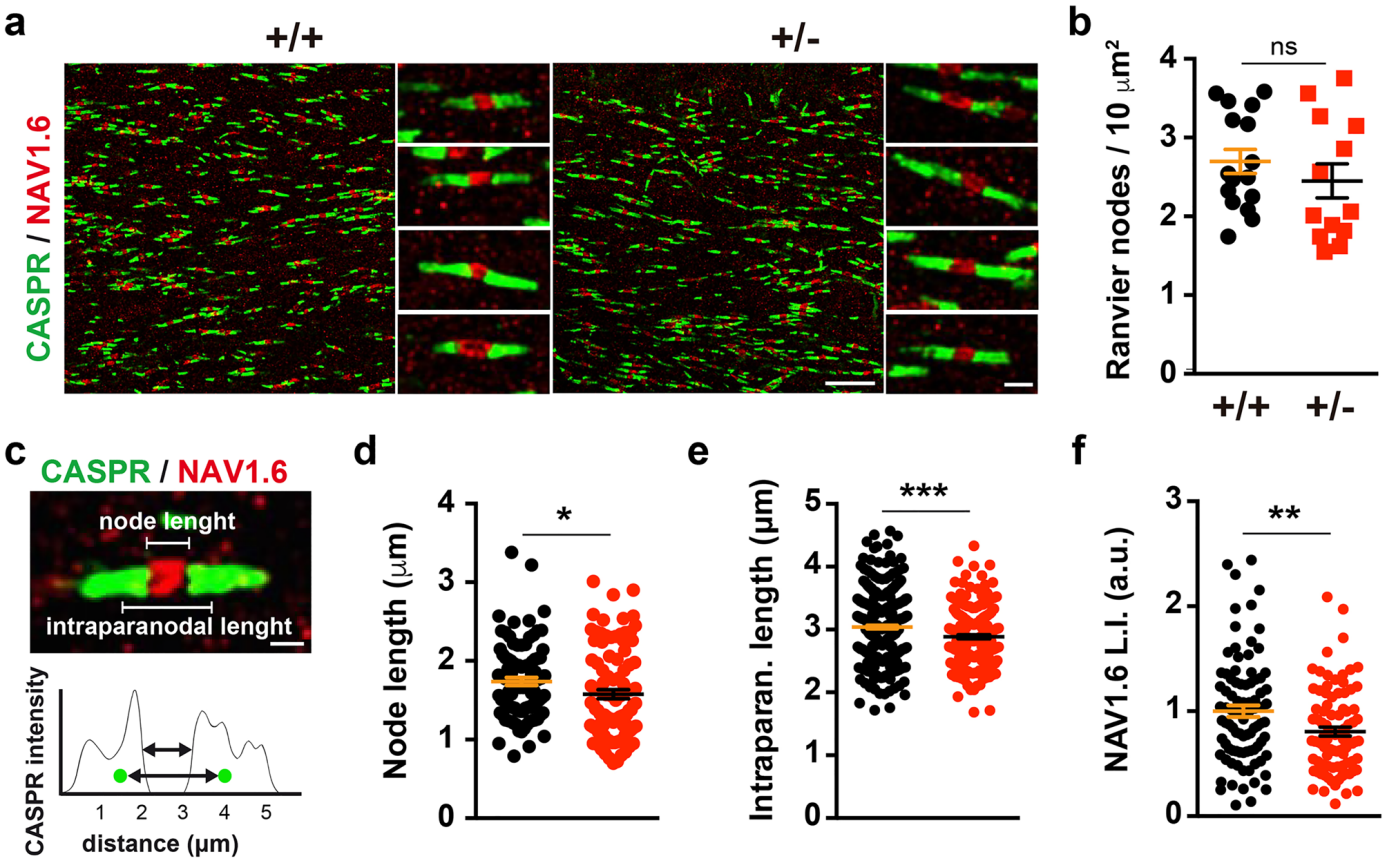
Alterations to the nodes of Ranvier in adult *Dyrk1a*^+/−^ mice. (**a**) Representative images from the *corpus callosum* of 2-month-old *Dyrk1a*^+/+^ (+*/*+) and *Dyrk1a*^+/−^ (+ */*−) mice showing the nodes of Ranvier labelled for NAV1.6 (red) and the paranodes for CASPR (green). (**b**) Density (mean ± SEM) of the nodes of Ranvier. The values correspond to the node density obtained from 16 (+/+) and 13 (+/−) images: ns, not significant, Student’s t-test. (**c**) Image showing a node of Ranvier and the intensity of CASPR staining in the paranodes. Green dots in the intensity profile represent the center of mass for each paranode as determined by the 3D object reconstruction. (**d**–**f**) Scatter dot plots showing the node length (**d**), intraparanodal length (**e**) and NAV1.6 labelling index (L.I.) (**f**) for individual nodes of Ranvier (n = 2–3 animals each genotype, 1 litter): 95 (+/+) and 100 (+/−) nodes in **d**; 356 (+/+) and 217 (+/−) in (**e**); 88 (+/+) and 90 (+/−) in (**f**); *P < 0.05 and **P < 0.01, Mann–Whitney U test; **P < 0.001, Student’s t-test.

Deficient axon-oligodendroglia communication can affect myelin biogenesis and the formation and maintenance of the nodes of Ranvier, events that are crucial for the rapid propagation of the APs through saltatory conduction^50,51^. Therefore, the organization of the nodes and the flanking paranodes was examined in the CC of *Dyrk1a*^+/−^ mice by immunostaining for NAV1.6 (voltage-gated sodium channel Na_v_1.6), the major sodium channel in the node region^52^, and for the CASPR (contactin-associated protein) that concentrates in the paranodal region^53^. No differences in callosal axon node densities were observed between *Dyrk1a*^+/−^ mutants and control mice (Fig. 6a,b). However, axonal regions immunolabeled for NAV1.6 were shorter in *Dyrk1a*^+/−^ mice (Fig. 6c,d) and their labelling was significantly weaker (Fig. 6e,f), indicating that mutant nodes may contain fewer NAV1.6 channels that could affect the speed of AP propagation.

### Slower conduction velocities in the ***Dyrk1a***^+/−^***corpus callosum***

**Figure 7:**
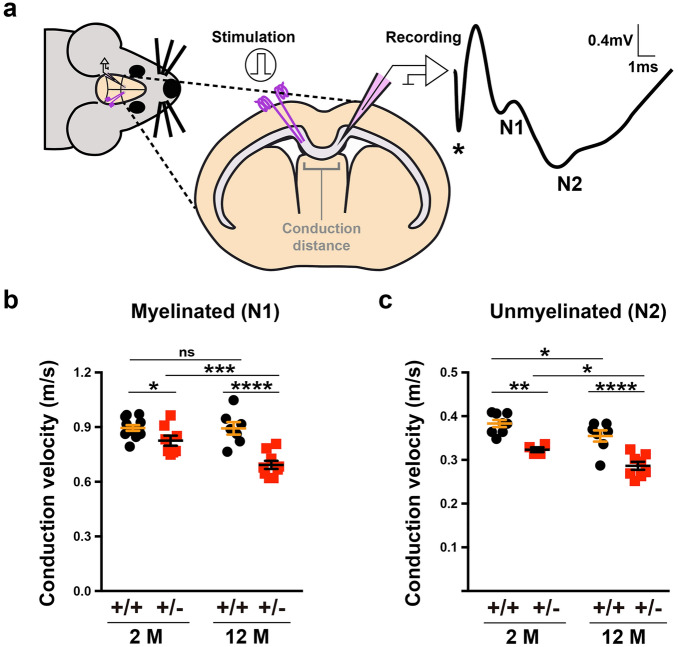
Slow velocities of action potential propagation in the *corpus callosum* of *Dyrk1a*^+/−^ mice in vivo. (**a**) Scheme of the electrophysiological recordings performed in 2- (2 M) and 12-month-old (12 M) *Dyrk1a*^+/−^ (+/−) and control (+/+) mice, and representative recording of compound action potentials (CAPs) arising from myelinated (N1) and unmyelinated (N2) axons. (**b**, **c**) Conduction velocities (mean ± SEM) for myelinated (**b**) and unmyelinated (**c**) axons in animals of the indicated age and genotype. n = 8–12 animals per genotype at 2 months (4 litters) and n = 7–9 animals per genotype at 12 months (3 litters): ns, not significant; *P < 0.05; **P < 0.01; ***P < 0.001 and ****P < 0.00, two-way ANOVA and Fisher’s LSD post-hoc test.

The abnormalities described so far in the CC of *Dyrk1a*^+/−^ mice indicate possible dysfunctions in AP propagation. To assess this possibility the conduction velocities of compound action potentials (CAPs) evoked by stimulation of callosal axons were measured in young (2-month-old) *Dyrk1a*^+/−^ and control mice (Fig. 7a). Conduction velocities arising from myelinated and unmyelinated axons were significantly slower in *Dyrk1a*^+/−^ mutants, although greater differences between the genotypes were evident in continuous rather than in saltatory conduction (slower velocities with respect to controls–myelinated axons 7.76 ± 3.29%, unmyelinated axons 15.63 ± 3.23%: see Fig. 7b,c). Hence, both myelin defects and axon dysfunction may contribute to the abnormal conduction velocities in *Dyrk1a*^+/−^ mutants.

Myelination continues at different rates throughout life to adapt brain circuits to diverse physiological needs^35^. Therefore, the same in vivo recordings were performed in aged (12-month-old) *Dyrk1a*^+/−^ and control mice, and like young animals continuous and saltatory conduction velocities were slower in older *Dyrk1a*^+/−^ mutants (myelinated axons 22.43 ± 4.43% and unmyelinated axons 19.27 ± 4.36%: see Fig. 7b,c). Notably, both continuous and saltatory conduction velocities decreased with age in *Dyrk1a*^+/−^ CCs, whereas only continuous conduction velocities were affected by age in control animals (Fig. 7b,c). This result indicates that defects in myelin homeostasis are enhanced with age in *Dyrk1a*^+/−^ mutants. Abnormalities in the CC would be expected to alter network synchronicity, thereby affecting higher brain functions^54^. Thus, the structural CC defects reported here probably contribute to the cognitive, language deficits and autistic traits in DYRK1A syndrome.

## Discussion

In this study we show that *DYRK1A* haploinsufficiency alters the production of cortical astrocytes and OPCs during development, defects that correlate with a delay in OPC differentiation in the CC and dysfunctions in AP propagation along callosal axons in adulthood.

The assembly of functional brain circuits relies on the generation of adequate numbers of neurons and glial cells at specific developmental times^33,34^. We previously identified alterations to the cortical neurogenic program of *Dyrk1a*^+/−^ embryos that produced an increase in neurons in all neocortical layers^12,27^, and here, we provide evidence that astrocyte generation is enhanced in the developing neocortex of *Dyrk1a*^+/−^ mice. Gliosis has been observed in magnetic resonance images of DYRK1A patients younger than 6-years-old^16^, suggesting that *DYRK1A* haploinsufficiency may also enhance glial cell production in humans. There is an increase of astrocytes in the frontal lobe in post-mortem DS foetal brains, indicating that trisomy 21 affects cortical astrogliogenesis^55^. Indeed, there is an increase in cortical astrocyte generation in the Ts1Cje mouse model of DS^39^, in which 67 mouse genes homologous to genes on human chromosome 21 are in trisomy, including *Dyrk1a*^56^. Notably, the overexpression of DYRK1A in this model upregulates the transcriptional activity of the pro-astrogliogenic factor STAT during the neurogenic-gliogenic switch, thereby promoting the differentiation of VZ progenitors into astrocytes^39^. In accordance with the role of DYRK1A in regulating STAT transcriptional activity, STAT3 activity was significantly reduced in the dorsal progenitors of *Dyrk1a*^+/−^ embryos (Supplementary Fig. S9). However, in vivo cell fate experiments showed that cortical *Dyrk1a*^+/−^ progenitors produce similar numbers of GFAP^+^ astrocytes to the wild-type mice. Therefore, the higher astrocyte densities in the *Dyrk1a*^+/−^ cerebral cortex don’t appear to be the result of enhanced neural progenitor differentiation but rather, to the surplus of progenitors at the onset of gliogenesis due to earlier developmental defects.

Evidence indicates that astroglial-derived signals regulate dendritic growth, synaptogenesis, and synapse pruning and maintenance^57,58^. Therefore, it is plausible that the excess of brain astrocytes contributes to the altered synaptic connectivity attributed to DYRK1A syndrome. In addition, DYRK1A influences neurogenesis, neuronal morphogenesis and glutamate neurotransmission, as it can interacts with key post-synaptic proteins and it modulates the membrane trafficking of synaptic vesicles at the pre-synaptic terminal^22,59,60^. At the synapse, astrocytes modulate neurotransmitter release by presynaptic terminals and the fidelity of excitatory neurotransmission by clearing glutamate and K^+^ ions from the synaptic cleft^61,62^. DYRK1A is expressed by the astrocytes in both human and mouse developing brains^46,63^. Thus, in addition to the excess of astrocytes, a dysfunction in these glial cells may also contribute to the neuropathology of DYRK1A syndrome, as shown previously in other ID and ASD disorders like Down’s, Rett’s and Fragile X syndromes^31,64^.

In the mammalian brain the CC is the principal axonal tract for interhemispheric communication. It facilitates higher-order cortical functions including language, intellectual processing, control of social and emotional stimuli, and the coordination of sensory-motor responses^65^. Alterations to the size of the CC and intracortical functional connectivity are common features of autism^66,67^. Abnormalities in the CC have been reported in patients with DYRK1A syndrome^19^ and other syndromic forms of ID/ASD, usually accompanied by language problems, such as in Angelman syndrome^68^, and FOXG1^69^ and CHD8-related syndromes^70^. The origin of white matter connectivity defects in ASD is not yet understood, although transcriptome data from animal models and post-mortem human tissue has recently pointed to dysfunctions in oligodendroglial cells and myelin-associated pathways^32^. In line with this, the chromatin remodelling factor CDH8 encoded by the gene mutated in CHD8-related syndrome is essential for oligodendrocyte development and repair^70^. Moreover, an oligodendrocyte targeted mutation in one *Chd8* allele causes similar deficits in CC myelination, AP propagation and social behaviour as that seen in the heterozygous *Chd8*^+/−^ mouse model^71^. The generation of the OPCs that first populate the developing CC is impaired in *Dyrk1a*^+/−^ mice, a defect correlated with a delay in the arrival of OPCs to the developing CC and in their differentiation into mature oligodendrocytes. OPC differentiation depends on a cell-intrinsic timer that defines the number of divisions before the cells exit the cell cycle and undergo differentiation^72^. Recent evidence indicates that this OPC timer is controlled by the interplay of several intrinsic factors with extracellular signalling molecules, and by neuronal activity^50,73^. Thus, the delay in oligodendroglial development in the *Dyrk1a*^+/−^ mouse is likely to be the result of the early deficit in OPC production as well as changes to the extrinsic cues provided by neurons and astrocytes.

The high density of axons (axons with smaller diameters) and the low number of myelinated axons are the most salient ultrastructural abnormalities to the CC in young *Dyrk1a*^+/−^ mice. The insolation provided by myelin is the main determinant of the speed of electrical signal propagation, the second being the diameter of the axon^51^. After the first synapse is formed, the cross-sectional area of long-range axons increases rapidly (radial axon growth)^74^. Thus, impaired radial axon growth is likely to be the basis of callosal hypomyelination and slow AP propagation in *Dyrk1a* haploinsufficient mice. There is significant evidence that the neuronal cytoskeleton is essential for axon development and function^75^. During the rapid phase of radial growth, neurofilaments (NFs) formed by the assembly of NF proteins (NFL, NFM and NFH) accumulate and they become the most abundant cytoskeletal component of the axon. Notably, during the first postnatal week there is a significantly reduction in the expression of the genes encoding NFL (*Nefl*) and NFM (*Nefm*) in the cerebral cortex of *Dyrk1a*^+/−^ mice^12^. NFL and NFM selectively regulate the axonal transport of neurofilament proteins and other cytoskeleton elements that promote radial axon growth^76^. Thus, changes in cytoskeleton composition during axonal development due to the abnormal levels of neurofilament proteins, as well as alterations to the neuronal actin and microtubule cytoskeleton as a result of the changes to the phosphorylation status of the DYRK1A substrates N-WASP, MAPB1 and β-Tubulin^22^, could underlie the formation of the small calibre callosal axons in the *Dyrk1a*^+/−^ mutant brains.

The correct function of saltatory conduction requires clustering of Na^+^ and K^+^ channels at the node of Ranvier. The recruitment and clustering of these ion channels relies on the precise organization of cytoskeleton scaffolding proteins^77,78^. Thus, alterations to the axonal cytoskeleton might also cause a shortening and a decrease in the NAV6.1 channel density at the nodes of Ranvier in *Dyrk1a*^+/−^ mutant brains. Once an axon is myelinated, the patterning of myelin changes in response to neuronal activity, adding new myelin sheaths and remodelling the existing ones^35,73^. In *Dyrk1a*^+/−^ neocortices, there is an increase in excitatory neurons and synapses and a decrease in proteins related to the GABAergic system^12,79^. Thus, disturbances in neuronal activity may also influence myelination in *Dyrk1a* haploinsufficient mice. Defects in myelination have been observed in other ID syndromes^80,81^, and recent evidence shows that myelination and myelin remodelling can be modulated by social experience and activity-dependent processes, which critically influences learning, memory and behaviour^54,82^. Therefore, it is likely that defects in both developmental myelination and adaptive experience-dependent myelination contribute to the neurological dysfunction evident in DYRK1A syndrome.

In addition to structural white matter abnormalities, there is an increase in PLP in the cerebral cortex of *Dyrk1a*^+/−^ mutants, the accumulation of which could result from defects in the membrane trafficking of PLP or to the neuronal signals that orchestrate this trafficking^50^. The accumulation of PLP in humans and mice caused by duplication of the *PLP1* gene leads to progressive demyelination^83^. Thus, the elevated PLP in *Dyrk1a*^+/−^ mice might also affect myelin integrity in the long-term, thereby contributing to the deficit in AP propagation along myelinated axons with age.

Myelination increases rapidly in the human cerebral cortex from birth until around 10 years of age, although it continues at a lower rate until the third decade of life^84^. Thus, therapies aim at improving myelination and axon conduction velocities during development and in adulthood could help ameliorate or revert the cognitive, social and language deficits in DYRK1A syndrome.

## Methods

### Mice

*Dyrk1a*^+/−^ embryos, postnatal and adult mice, and their control *Dyrk1a*^+/+^ littermates were generated from crosses between C57BL/6;129S2 *Dyrk1a*^+/−^ males and wild-type females and genotyped as described elsewhere^27^. These crosses produced an average of 8 littermates. The day of the vaginal plug was defined as E0.5 and the day of birth was defined as P0. After weaning, mice from the same litter and gender were housed in groups, with food and water supplied ad libitum. Studies in embryos and postnatal animals were performed in both sexes. In adult animals, immunofluorescence was performed on female tissue, electron microscopy on male tissue, and in vivo electrophysiology on both males and female mice.

### Tissue preparation and immunostaining

Embryos were recovered at the times indicated and their heads were fixed overnight at 4 °C by immersion in 4% paraformaldehyde (PFA). Postnatal and adult mice were anesthetized deeply in a CO_2_ chamber and perfused transcardially with PFA at room temperature (RT). The brain of each embryo or animal was removed, post-fixed and embedded in agarose to obtain serial free-floating vibratome Sects. (50 μm: Leica VT1000S). For immunofluorescence, the sections were incubated for 1 h at RT in phosphate buffered saline (PBS, pH 7.4) containing 0.2% Triton X-100 and 10% foetal bovine serum (FBS), and probed for 48 to 72 h at 4 °C with the primary antibodies diluted in antibody buffer (PBS with 0.2% Triton X-100 and 5% FBS). The primary antibodies used were: goat anti-SOX2 (1:250, Cat# sc-17320, RRID AB_2286684: Santa Cruz Biotechnology); mouse anti-CASPR (1:200, Cat# 75–001, RRID AB_2083496: NeuroMab), anti-APC (1:200, clone CC-1, Cat# OP80, RRID AB_2057371: Millipore) and anti-DYRK1A (1:250, Cat# H00001859-M01, RRID AB_534844: Abnova); rabbit anti-GFAP (1:500, Cat# Z0334, RRID AB_10013382: DAKO), anti-IBA1 (1:600, Cat# 019–19,741, RRID AB_839504: Wako), anti-NAV1.6 (1:200, Cat# ASC009, RRID AB_2040202: Alomone Labs), anti-OLIG2 (1:500, Cat# ab9610, RRID AB_570666: Millipore) and anti-SOX9 (1:500, a kind gift from Dr Wegner, Friedrich-Alexander-Universität Erlangen-Nürnberg, Germany); rat anti-MBP (1:500, Cat# MAB386, RRID AB_94975: Millipore) and anti-PDGFRα (1:100, Cat# 558,774, RRID AB_397117: BD Biosciences). The sections were then washed and the primary antibodies were detected using Alexa-565, Alexa-488 or Alexa-633 conjugated secondary antibodies (1:1000: Life Technologies). Cell nuclei were stained with 4′,6-diamidino-2-phenylindole (DAPI: Sigma-Aldrich). The specificity of the immunoreactions was assessed by omitting the primary antibody.

### Image acquisition and cell counts

Images from coronal brain sections were acquired on: a Leica AF7000 motorized wide-field microscope using a 5 × or 10 × objective and a digital CCD camera; on a SP5 confocal microscope using an HCX PL APO 40x/1.25–0.75 OIL CS objective; or on a Zeiss LSM-780 confocal microscope using a 25 × multi immersion objective. Fiji software was used to obtain the maximal projections from the z-stack images and for image merging, resizing and cell counting. The counting of OLIG2^+^ and PDGFRα^+^ cells in embryos was performed in a 200 μm wide column of the dorso-lateral cortical wall, or in a 150 × 300 μm rectangle of the LGE. GFAP^+^ and SOX9^+^ cells were counted in 500 μm or 1000 μm wide columns of the somatosensory barrel cortex of postnatal and adult brains. Unless otherwise stated, OLIG2^+^ and CC1^+^ (Adenomatous polyposis-APC) cells were quantified in the centre of the CC. The total cells were quantified based on DAPI staining. Cell counts in the adult CC and somatosensory cortex were estimated using Fiji software from serial sections spanning from Bregma + 1.10 mm to − 1.82 mm^85^. All counts were carried out on 2 to 8 images obtained from 3 to 17 embryos/animals per experimental condition.

### Density and length of the Nodes of Ranvier

Coronal sections at the level of the CC were stained for NAV1.6 and CASPR, and images were acquired with a Zeiss LSM-780 microscope. Only nodes with the NAV1.6 labelled domain flanked by CASPR labelled domains were considered in the analysis. To estimate the node densities, we used a 40 × oil DIC objective, a 3 × optical zoom and 15 μm thick z-stacks, processing the images were with the Fiji software. A 63 × oil DIC objective and 45 μm thick z-stacks, with 0.15 μm z-steps, were used to estimate node and intraparanodal lengths. Deconvolution of the images was performed with the Huygens Professional Deconvolution Software (https://svi.nl/Huygens-Software) and the deconvoluted stacks were then processed with the 3D Object Counter Plug-in of Fiji. The intraparanodal length was calculated as the Euclidean distance between two CASPR labelled paranodes separated by a NAV1.6 labelled node according to the following equation:

$$L = \sqrt {\left( {x_{b} - x_{a} } \right)^{2} + \left( {y_{b} - y_{a} } \right)^{2} + \left( {z_{b} - z_{a} } \right)^{2} }$$

where (x, y, z) were the three-dimensional coordinates of the centre of masses of a paranode. We selected labelled nodes with the two labelled paranodes in at least eight z-steps to calculate the node length. The maximum intensity projection of all the slices was generated and the intensity profile plotted using the Plot Profile Command in Fiji. The nodes and node lengths were quantified in images from 4–5 animals, and from 2 to 3 animals per genotype, respectively.

### Electron microscopy

Anesthetized P19 and P60 mice were perfused transcardially with 4% PFA and 2% glutaraldehyde in PBS at RT. Their brains were removed and post-fixed in the same fixative at 4 ℃ for 24 h, and then segmented in a medial sagittal plane and trimmed into 2–3 mm thick segments. Sagittal slices were washed in PBS, stained with 1% OsO_4_, dehydrated in graded concentrations of ethanol and embedded in Epon 812 (Electron Microscopy Science). Serial 0.5 μm semi-thin sections were stained with toluidine blue and examined on a Leica DMB microscope. Thin Sects. (60–80 nm) were obtained with a Reichert Ultracut ultramicrotome equipped with a diamond knife, stained with uranyl acetate and lead citrate, and examined on a JEOL JEM-1001 electron microscope. Digital images were acquired with a MegaViewIII camera, and the image fields were analysed using the Fiji software and with a modified version of software designed to study axonal morphometry^86^. For the morphometric evaluation of axons, fibres were examined at 11,000 × magnification. Only fibres with microtubules and neurofilaments sectioned perpendicular to their longitudinal axes were selected for analysis. For small axons and thinly myelinated fibres, the boundary between the nerve fibres and the surrounding background was defined by a semi-automatic method. The axonal contour and the external contour of the myelin sheath (fibre contour) were traced manually on enlarged images. A set of custom macros allowed the length of a line, the cross-sectional area, and the lengths of the major and minor axes of the best fitting ellipse to be calculated. Fibre and axonal diameters were deduced from the fibre and axonal perimeters, assuming a cylindrical shape of the axons. These basic data were used to derive the g-ratio and myelin sheath thickness^86^. Electron microscopy images obtained from a minimum of three animals per experimental conditions were included in the analysis.

### Electrophysiology

Electrophysiological recordings were obtained from *Dyrk1a*^+/−^ and *Dyrk1a*^+/+^ mice at two different ages: 2- and 12-months. Surgical anaesthesia was induced by intraperitoneal injection of a cocktail of ketamine (80 mg/Kg) and xylazine (10 mg/Kg). The depth of anaesthesia was evaluated by continuously monitoring the response to paw pinching and through electrocorticogram recordings, and a supplementary dose of ketamine (40 mg/Kg) was administrated if necessary. The animal’s body temperature was maintained using a heating pad (Kent Scientific, Torrington, CT, USA) during the surgical procedure. Mice were placed in a stereotaxic device and two craniotomies were drilled to access the CC, and to allow insertion of the recording and stimulation electrodes on each side of the midline (Bregma positions: 0.98–1.1–1.5 in *Dyrk1a*^+/+^ mice and 0.8–1.0–1.4 in *Dyrk1a*^+/−^ mice). Ten consecutive electrical rectangular pulses (100 µs length and 800 µA intensity) were delivered at 1 s intervals through bipolar stainless steel electrodes, and the neural activity of the callosal CAPs generated in response to the stimulation were recorded through tungsten microelectrodes with a 12 MΩ resistance (AM Systems, Sequim, WA). The evoked callosal CAPs were amplified and filtered at a frequency band-pass of 0.1–1000 Hz (AM-systems Differential AC amplifier 1700) and at an additional 50 Hz specific filter (Hum Bug, Digitimer, Welwyn Garden City, UK), digitized through an analogue-to-digital interface (ADInstruments, PowerLab 4/25 T, Dunedin, New Zealand), and the Scope v3.7.3 (ADInstruments, PowerLab) software was used to acquire and average 10 consecutive CAP traces in response to stimulation. LabChart Reader v.8 software was used to analyse the neuronal activity offline. Latencies to the maximal amplitude of 1^st^ (N1) and 2^nd^ (N2) negative CAP peaks were measured, and conduction velocities were assessed according to the distance between the stimulation and recording electrodes.

### Statistical analysis

Statistical analyses were performed with GraphPad Prism v5.0a (GraphPad Software). The normal distribution of the values was assessed by the D’Agostino-Pearson omnibus normality test. Significance was assessed with a 2-tailed unpaired Student’s *t*-test, a two-way ANOVA and a Fisher’s LSD post-hoc test for normally distributed data, or with a 2-tailed unpaired Mann–Whitney U test for non-normally distributed data. Differences were considered significant at *P* < 0.05. The data are represented as the mean ± standard error of the mean (SEM), unless indicated in the figure legend.

### Ethics approval

Experiments with mice were carried out in accordance with the European Union guidelines (Directive 2010/63/EU), and all the protocols were approved by the Ethics Committees of the CSIC, Parc Científic de Barcelona (PCB) and University Miguel Hernandez, and by the appropriate local government bodies. This study is reported in accordance with ARRIVE guidelines.

## Supplementary Information

Supplementary Information.

## Data Availability

The datasets obtained and analysed in the current study are available from the corresponding authors on reasonable request.
