# Supplementary material for: Impaired macroglial development and axonal conductivity contributes to the neuropathology of DYRK1A-related intellectual disability syndrome
Source: Sci Rep. 2022 Nov 19;12:19912. doi: 10.1038/s41598-022-24284-5 (PMC9675854; doi:10.1038/s41598-022-24284-5)
Supplement: Supplementary file 1 — Supplementary Information. [file 41598_2022_24284_MOESM1_ESM.pdf]

Impaired macroglial development and axonal conductivity contributes to the neuropathology of *DYRK1A*-related intellectual disability syndrome

Isabel Pijuan, Elisa Balducci, Cristina Soto-Sánchez, Eduardo Fernández, María José Barallobre, Maria L. Arbonés

Supplementary Methods
Supplementary References
Supplementary Figures 1 to 10

Supplementary Methods

In-utero electroporation

Electroporation was performed in the dorsal telencephalon of E16.5 mouse embryos as described elsewhere [1]. Glial progeny were analysed in the cortical dorso-lateral region of electroporated P5 mice. The pPB-CAG-GFP vector and the pCyL43-PBase *piggyBac* tansposase expression vector (pCAG-PBase) were used to express the enhanced Green Fluorescence Protein (eGFP) [2] in all progeny of the electroporated progenitors. STAT3 activity was analysed in the dorsal telencephalon of embryos electroporated with the Luciferase reported plasmids GFL1-pGL3 or GL1-S-pGL3 [3] and a *Renilla* luciferase reporter used for normalization. The plasmid DNA (1.5 μg, equal weight of pPB-CAG-GFP and pCAG-PBase, and 30:1 ratio for GFL1-pGL3/GL1-S-pGL3 and the *Renilla* plasmid) diluted in PBS containing Fast Green FCF (1 mg/ml: Sigma-Aldrich) was injected into the lateral ventricle with a fine glass micropipette. Five electrical pulses of 50 ms and 38 V at an interval of 950 ms were then delivered using 5 mm diameter electrodes (CUY650P5, Nepagene). For Luciferase assay, embryos were harvested 24h after electroporation. The electroporated telencephalic region was carefully dissected and homogenized in the Dual-Luciferase Assay System (Promega). Firefly and Renilla luciferase activities were measured in an Orion II Microplate Luminometer (Berthold).

Western blotting

Cerebral hemispheres were homogenized in 25 mM Tris-HCl [pH 7.4] containing 1 mM EDTA, 1% SDS and protease inhibitors, and the total protein extracts (≈40 μg) were resolved by SDS-PAGE following standard procedures, transferred to nitrocellulose membranes (Hybond-ECL, Amersham Biosciences) and probed with primary antibodies: mouse anti-GAPDH, 1:2000 (Cat# MAB374, RRIP AB_2107445: Millipore); rabbit polyclonal anti-PLP, 1:1000 (Cat# NB100-74503, RRIP AB_1049196: Novus Biologicals); and a rat monoclonal anti-MBP, 1:500 (Cat# MAB386, RRIP AB_94975: Millipore). Antibody binding was detected by infra-red fluorescence using the LI-COR Odyssey IR Imaging System V3.0 (LI- COR Biosciences). GADPH was use for the normalization of MBP (Myelin Basic Protein) and PLP (Proteolytic Protein) levels in brain extracts from 11 animals per genotype (4 females and 7 males).

RNA extraction and RT-qPCR

RNA from the brain hemispheres was extracted using the TriPure isolation reagent (Roche) according to the manufacture's instructions and cDNAs were synthesized from 1 μg of total RNA using the cDNA synthesis kit (Roche), following the manufacturer's

indications. Real-time qPCR (RT-qPCR) was carried out on the Lightcycler 480 platform (Roche) using EvaGreen Dye (Niborlab) and with *Peptidyl-prolyl isomerase* (*Ppia*) as the reference gene for data normalization. The PCR primers used were: *Mbp* forward (5'– CTTCAAAGACAGGCCCTCAG – 3'), reverse (5'– CCAGGTACTTGGATCGCTGT – 3'); *Plp1* forward (5'– TTGGCGACTACAAGACCACC – 3'), reverse (5'– AGCCATACAACAGTCAGGGC – 3'); and *Ppia* forward (5'– ATGGCAAGACCAGCAAGAAG – 3'), reverse (5'– TTACAGGACATTGCGAGCAG – 3'). The cDNAs studied were obtained from 3-4 brains per genotype and each sample was assayed in triplicate. The Ct (threshold cycle) was calculated using the relative quantification of the second derivative maximum method with the Lightcycler 480 1.2 software (Roche).

References

1 Saito, T. & Nakatsuji, N. Efficient gene transfer into the embryonic mouse brain using in vivo electroporation. *Dev Biol* **240**, 237-246, doi:10.1006/dbio.2001.0439 (2001).

2 Nagy, K. *et al.* Induced pluripotent stem cell lines derived from equine fibroblasts. *Stem Cell Rev Rep* **7**, 693-702, doi:10.1007/s12015-011-9239-5 (2011).

3 Nakashima, K. *et al.* Synergistic signaling in fetal brain by STAT3-Smad1 complex bridged by p300. *Science* **284**, 479-482, doi:10.1126/science.284.5413.479 (1999).

Figures

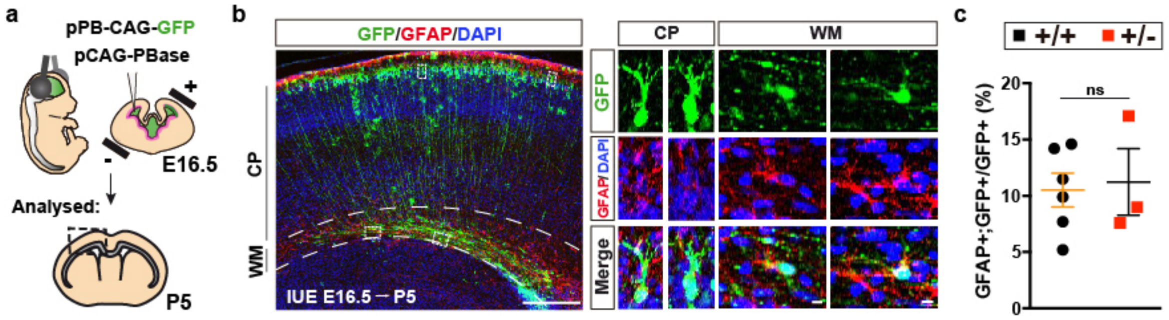

Fig. S1. Differentiation of dorsal *Dyrk1a*$^{+/-}$ neural progenitors into astroglial cells. (**a**) Schematic representation of the *in-utero* electroporation of E16.5 *Dyrk1a*$^{+/+}$ (+/+) and *Dyrk1a*$^{+/-}$ (+/-) embryos to evaluate the astroglial competence of dorsal ventricular zone progenitors. The electroporated plasmids containing the *Piggybase* transposase (pCAG-PBase) and transposon-specific inverted terminal repeat sequences (pPB-CAG-GFP) are indicated. (**b**) Representative coronal section from a P5 control brain immunostained for GFAP (red) and with the nuclei labelled by DAPI (blue) showing the astroglia progeny (GFAP$^+$;GFP$^+$ cells) of the electroporated progenitors in the cortical plate (CP) and white matter (WM, defined by the dashed lines). The right panels show magnifications of representative double-labelled cells (boxed cells) in the CP and WM. Scale bars: 500 μm and 10 μm (magnification panels). (**c**) Percentage (mean ± SEM) GFP$^+$ cells expressing GFAP (GFAP$^+$;GFP$^+$ cells/total GFP$^+$ cells). The values correspond to individual embryos (1 litter): ns, not significant, Student's t test.

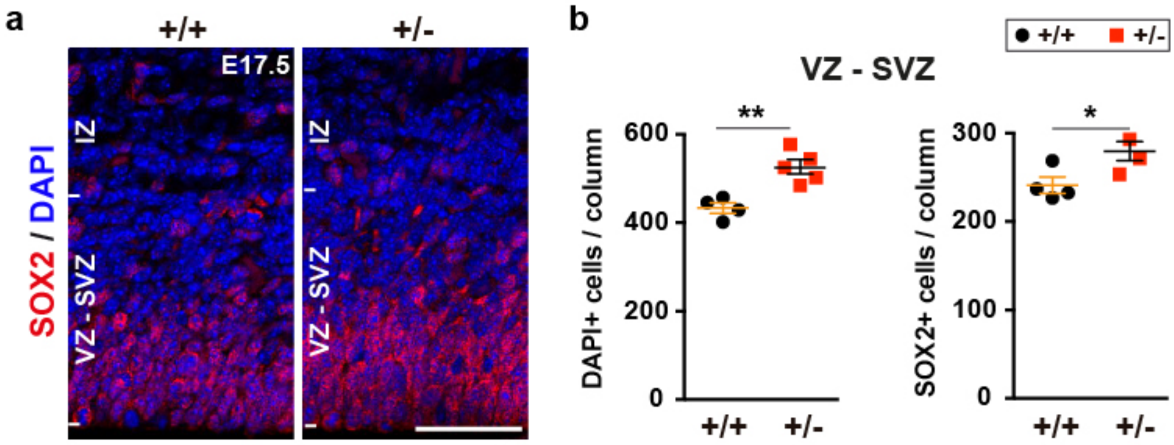

Fig. S2. The number of neural progenitors in the dorsal proliferative region of *Dyrk1a*$^{+/-}$ brains during the neurogenic/gliogenic switch. (**a**) Representative images of the dorsal telencephalon of E17.7 *Dyrk1a*$^{+/+}$ (+/+) and *Dyrk1a*$^{+/-}$ (+/-) brains stained for SOX2 (red), and with the nuclei labelled by DAPI (blue): VZ-SVZ, ventricular and subventricular zones; IZ, intermediate zone. (**b**) mean (± SEM) of the total cells (lefts) and SOX2 labelled cells (right) quantified in the ventricular and subventricular zones (VZ-SVZ) of 200 μm wide columns. The values correspond to individual embryos (1 litter): **P* <0.05 and ***P*<0.01, Student's t-test. Scale bar: 50 μm.

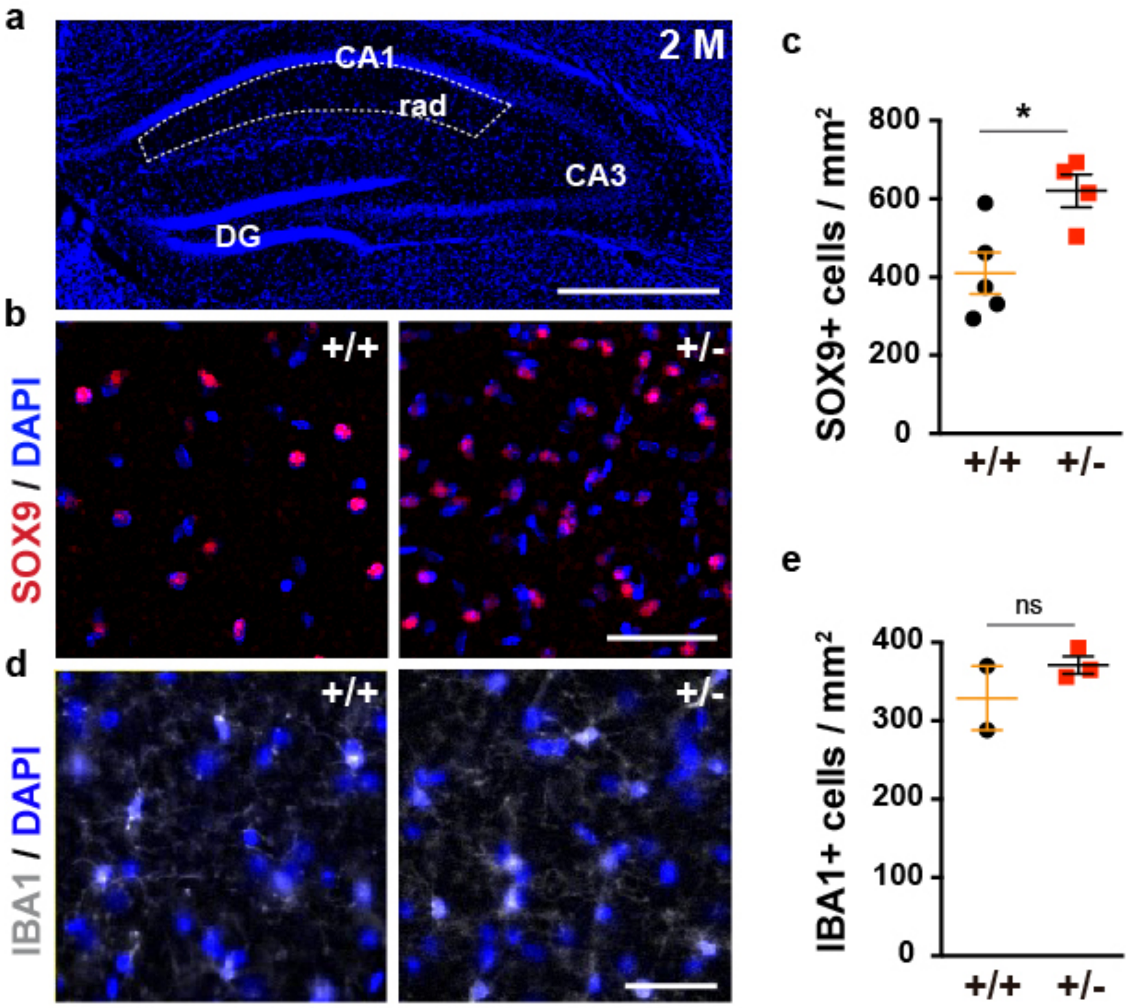

Fig. S3. Increased numbers of SOX9 labelled astrocytes in the hippocampus of adult *Dyrk1a*$^{+/-}$ mice. (**a**) Image of a coronal brain section of a *Dyrk1a*$^{+/+}$ (+/+) hippocampus with the nuclei labelled by DAPI (blue). The dashed lines define the *stratum radiatum* layer (rad): CA1, CA (*cornu ammonis*) area 1; CA3, CA area 3; GD, *dentate gyrus*. (**b**,**d**) Representative images of the rad of 2-month-old +/+ and *Dyrk1a*$^{+/-}$ (+/-) mice stained for SOX9 (red, **b**) and IBA1 (grey, **d**), and with the nuclei labelled with DAPI. (**c**,**e**) Density (mean ± SEM) of SOX9^{+} (**c**) and IBA1^{+} cells (**e**) in the rad of +/+ and +/- brains. The values correspond to individual animals (2 litters in **c** and 1 litter in **e**), and were obtained by counting cells in squares of approximately 0.25 mm^2: ns, not significant; *P<0.05, Mann Whitney U test. Scale bar: 500 µm (**a**) and 50 µm (**b**).

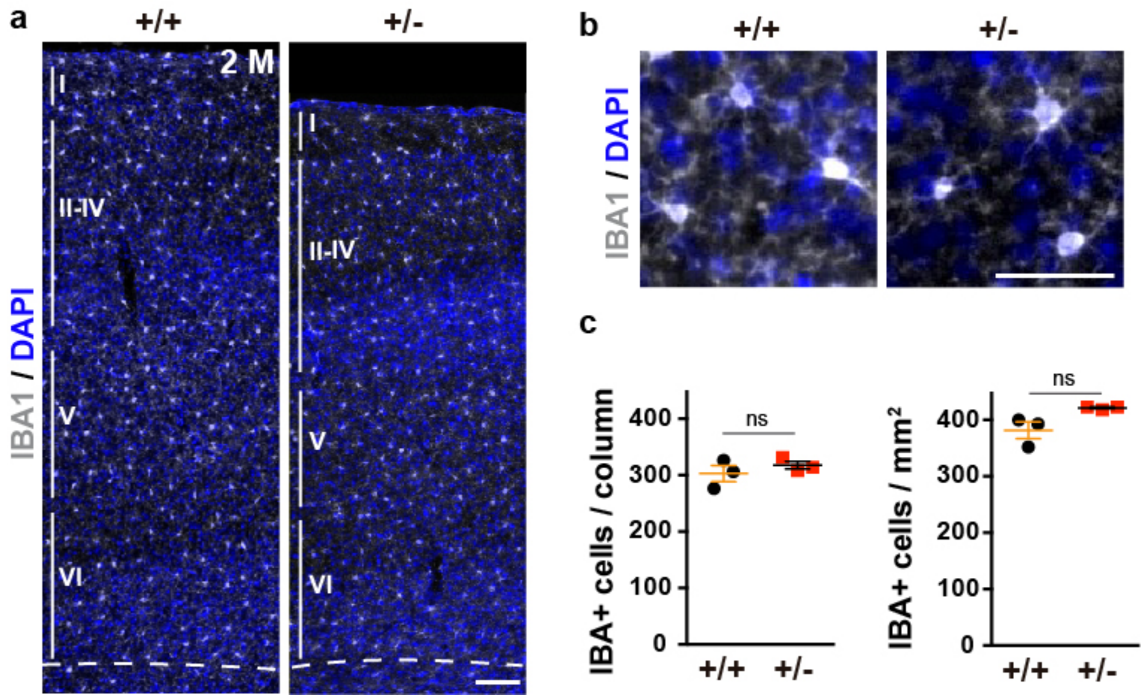

Fig. S4. Normal expression of the IBA1 microglia marker in the cerebral cortex of adult *Dyrk1a*$^{+/-}$ mice. (**a**) Representative images of the neocortex of 2-month-old *Dyrk1a*$^{+/+}$ (+/+) and *Dyrk1a*$^{+/-}$ (+/-) animals stained for IBA1 (grey) and with the nuclei labelled with DAPI (blue) indicating the position of layers I to VI. (**b**) Magnifications showing the staining pattern of cortical IBA1+ cells. (**c**) Mean number (± SEM, left) and mean density (± SEM, right) of IBA1^{+} cells quantified in 500 µm wide columns from layers I to VI. The values correspond to individual animals (1 litter): ns=not significant, Student's t-test. Scale bar: 100 µm (**a**) and 50 µm (**b**).

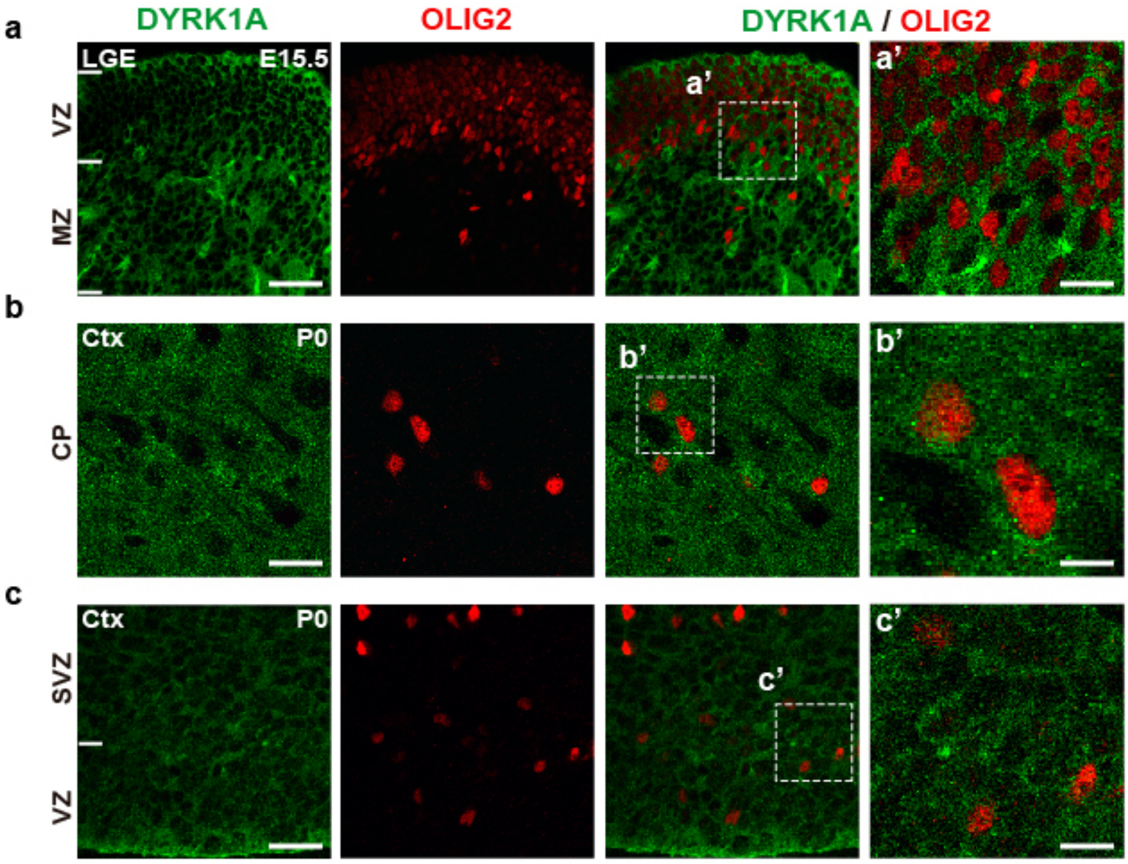

Fig. S5. DYRK1A is expressed in ventral and dorsal oligodendroglial precursor cells. (**a**) Representative images of the lateral ganglionic eminence (LGE) of E15.5 embryos stained for DYRK1A (green) and OLIG2 (red): VZ, ventricular zone; MZ, marginal zone. Magnification of boxed areas (**a’**) shows OLIG2^{+} progenitors delaminating from the VZ and expressing DYRK1A. (**b**, **c**) Representative images of the cortical plate (CP in **b**) and proliferative regions (ventricular and subventricular zones: VZ and SVZ in **c**) in the dorsal telencephalon of P0 mice stained for DYRK1A and OLIG2. **b’** and **c’** are magnifications of the dashed box areas. Scale bar: 150 μm (**a**), 50 μm (magnification **a’**); 25 μm (**b** and **c**), 50 μm (magnification **b’** and **c’**).

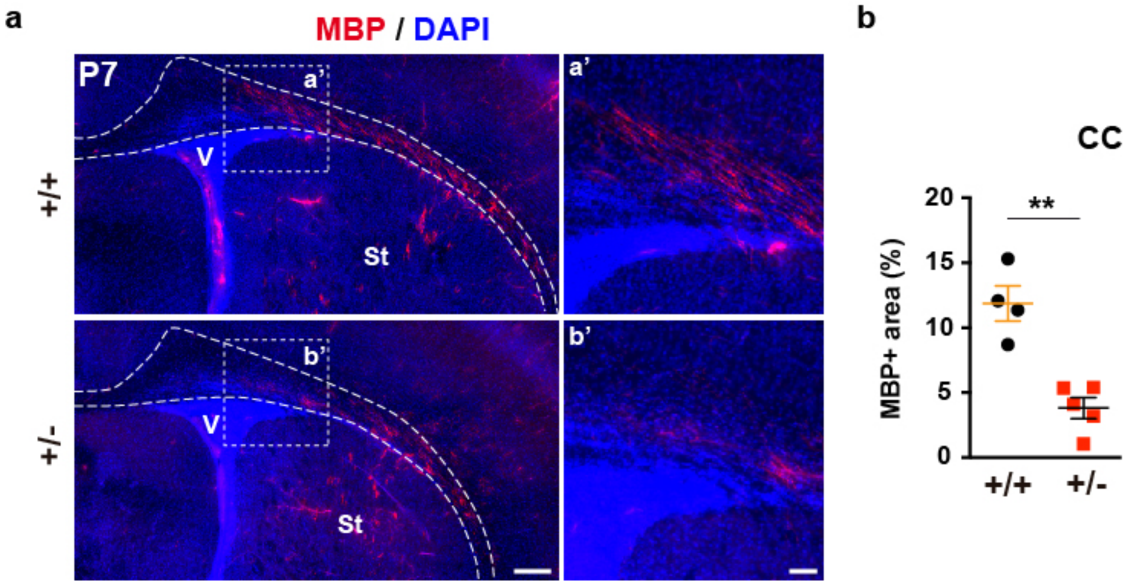

Fig. S6. Reduced myelin basic protein (MBP) expression in the corpus callosum of postnatal *Dyrk1a*$^{+/-}$ mice. (**a**) Representative images of P7 *Dyrk1a*$^{+/+}$ (+/+) and *Dyrk1a*$^{+/-}$ (+/-) coronal brain sections stained for MBP (red), with the nuclei labelled by DAPI (blue), and magnifications of the dashed box areas in **a'** and **b'**. Dashed lines define the *corpus callosum*: St, striatum. (**b**) Percentage (mean ± SEM) of the area of the CC labelled for MBP in +/+ and +/- sections. The values correspond to individual animals (1 litter): **$P<0.01$, Student's t-test. Scale bar: 25 μm (**a**); 50 μm (magnifications **a'** and **b'**).

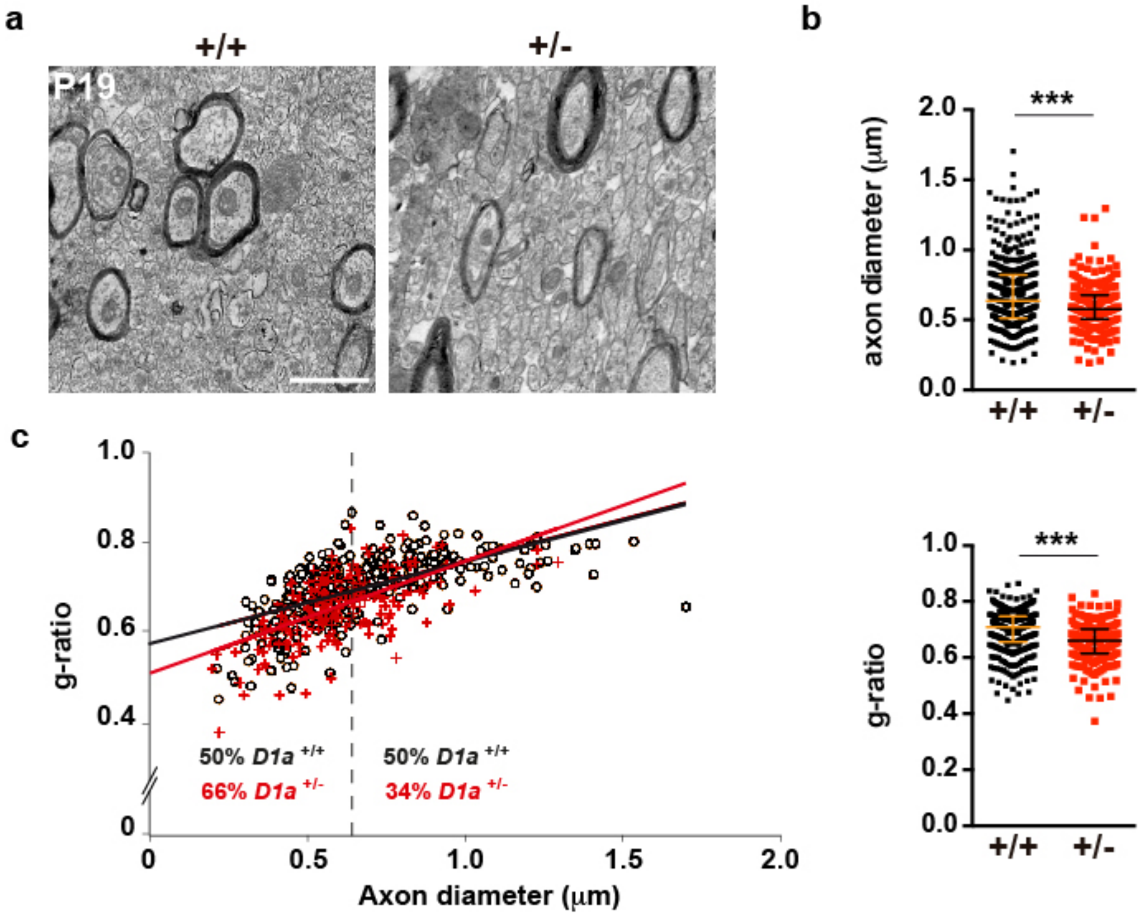

Fig. S7. Ultrastructural alterations to the *corpus callosum* of postnatal *Dyrk1a*$^{+/-}$ mice. (**a**) Representative electron micrographs of the P19 corpus callosum in +/+ and +/- mice. Scale bar: 1 μm. (**b**) Median value of the axon diameter (± interquartile range) for each genotype. (**c**) Scatter plot of the g-ratios of individual fibres relative to their respective axon diameter. The dashed line indicates the median axon diameter in +/+ mice and the numbers on either side of this line reflects the percentage of axons below (left) or above (right) this value for each genotype, and the median value of the g-ratio (± interquartile range) for each genotype. The values in **b** and **c** were obtained from 398 (+/+) and 200 (+/-) axons: n=2-3 animals each genotype (2 litters). ****P*<0.001, Mann-Whitney test.

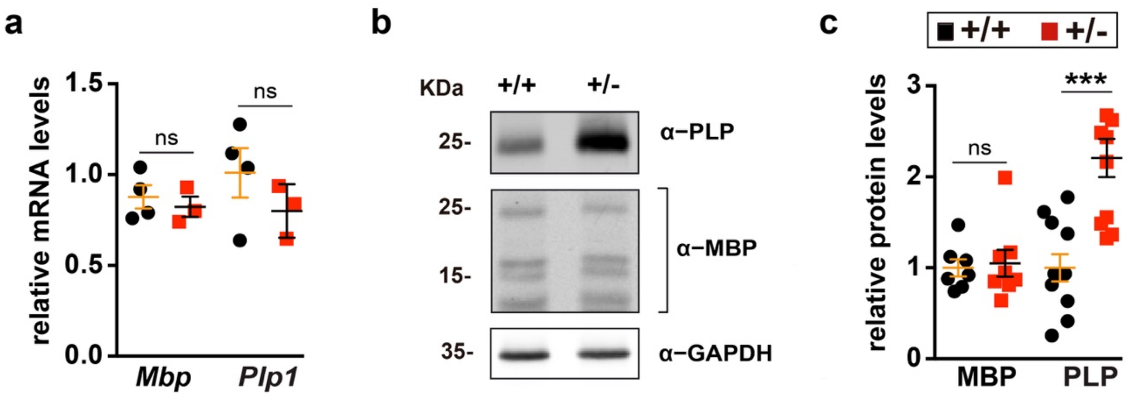

Fig. S8. Altered PLP levels in the cerebral cortex of adult *Dyrk1a*$^{+/-}$ mice. (**a**) *Mbp* and *Plp1* mRNA expression relative to the levels of *Ppia* in the cerebral cortex of 2-month-old (2 M) *Dyrk1a*$^{+/+}$ (*+/+)* and *Dyrk1a*$^{+/-}$ (*+/-)* mice arbitrarily set as 1. (**b**) Representative immunoblots of extracts prepared from the cerebral cortex of 2 M mice probed with the antibodies indicated. Image obtained with the LI-COR Odyssey IR Imaging System V3.0 (original in Fig. S10a), cropped and converted into a grayscale image in Fiji software. (**c**) The MBP and PLP protein was normalized to GAPDH and expressed relative to the controls, and the values (mean ± SEM) in **a** and **c** correspond to individual animals (1 litter in **a** and 3 litters in **c**). ns, not significant; ****P*<0.001, Student's t-test.

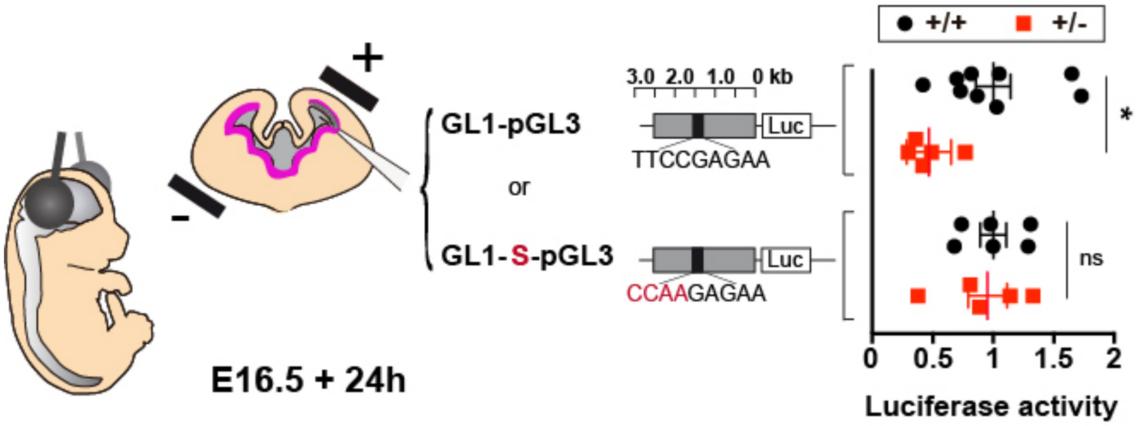

Fig. S9. Altered STAT3 activity in the dorsal telencephalon of *Dyrk1a*$^{+/-}$ embryos during the neurogenic/gliogenic switch. Scheme of the *in utero* electroporation experiment performed in E16.5 +/+ and +/- embryos, and of the luciferase (Luc) reporter plasmids used to evaluate STAT3 activity in apical progenitors. The grey boxes in these plasmids indicate the GFAP promoter sequences and the black boxes the STAT3 binding site. The mutated STAT3 binding site in GL1-S-pGL3 is indicated in red. The plot on the right shows the mean (± SEM) Luciferase values relative to the *Renilla* values in the dorsal telencephalon of +/+ and +/- embryos 24h after electroporation. WT values were arbitrarily set as 1 and the values correspond to individual embryos (2-3 litters each condition): ns=not significant; **P*<0.05, Student's t-test.

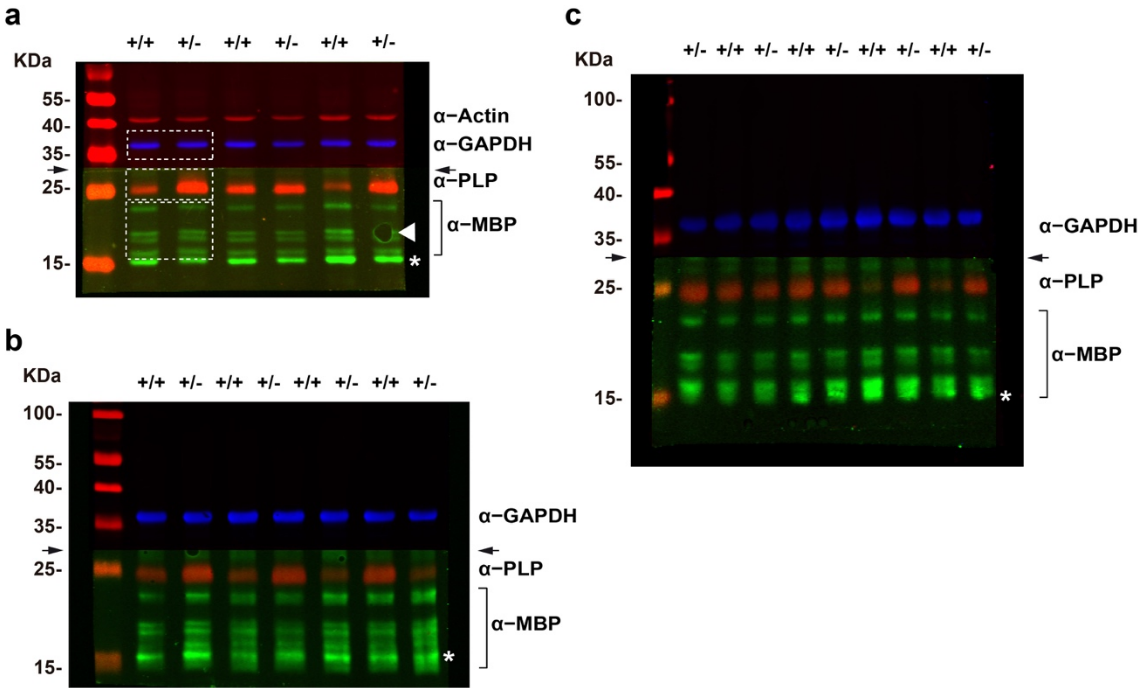

Fig. S10. Original immunoblots corresponding to protein quantifications in Fig. S8. (**a**-**c**) Asterisk indicates MBP unspecific bands. Arrows indicate the cutting place of the membrane for its hybridization with the indicated antibodies: the upper part of the membrane with anti-GAPDH (**a**-**c**) and -Actin (**a**); and the lower with anti- MBP and -PLP (**a**-**c**). Arrowhead in **a** points to a transfer bubble. This lane was excluded from the analysis. White squares indicate the cropped areas shown in Fig. S8b.
